# Toward an Understanding of the Propensity for Crystalline Hydrate Formation by Molecular Compounds. Part 2

**DOI:** 10.1021/acs.cgd.1c00353

**Published:** 2021-07-30

**Authors:** Rana Sanii, Ewa Patyk-Kaźmierczak, Carol Hua, Shaza Darwish, Tony Pham, Katherine A. Forrest, Brian Space, Michael J. Zaworotko

**Affiliations:** † Department of Chemical Sciences and Bernal Institute, University of Limerick, Co. Limerick Y94T9PX, Ireland; ‡ Department of Materials Chemistry, Faculty of Chemistry, Adam Mickiewicz University, Uniwerystetu Poznańskiego 8, 61-614, Poznań, Poland; § School of Chemistry, University of Melbourne, Victoria, 3010, Australia; ⊥ Department of Chemistry, University of South Florida, 4202 East Fowler Avenue, Tampa, Florida 33620, United States

## Abstract

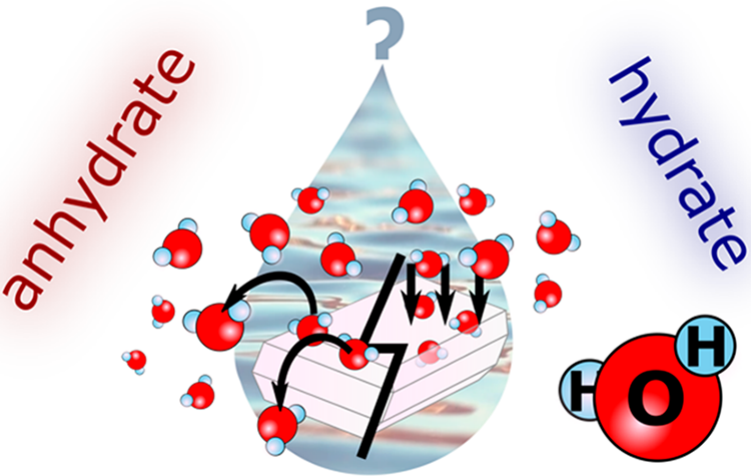

The propensity of molecular organic compounds to form stoichiometric or nonstoichiometric crystalline hydrates remains a challenging aspect of crystal engineering and is of practical relevance to fields such as pharmaceutical science. In this work, we address the propensity for hydrate formation of a library of eight compounds comprised of 5- and 6-membered *N*-heterocyclic aromatics classified into three subgroups: linear dipyridyls, substituted Schiff bases, and tripodal molecules. Each molecular compound studied possesses strong hydrogen bond acceptors and is devoid of strong hydrogen bond donors. Four methods were used to screen for hydrate propensity using the anhydrate forms of the molecular compounds in our library: water slurry under ambient conditions, exposure to humidity, aqueous solvent drop grinding (SDG), and dynamic water vapor sorption (DVS). In addition, crystallization from mixed solvents was studied. Water slurry, aqueous SDG, and exposure to humidity were found to be the most effective methods for hydrate screening. Our study also involved a structural analysis using the Cambridge Structural Database, electrostatic potential (ESP) maps, full interaction maps (FIMs), and crystal packing motifs. The hydrate propensity of each compound studied was compared to a compound of the same type known to form a hydrate through a previous study of ours. Out of the eight newly studied compounds (herein numbered **4**–**11**), three Schiff bases were observed to form hydrates. Three crystal structures (two hydrates and one anhydrate) were determined. Compound **6** crystallized as an isolated site hydrate in the monoclinic space group *P*2_1_/*a*, while **7** and **10** crystallized in the monoclinic space group *P*2_1_/*c* as a channel tetrahydrate and an anhydrate, respectively. Whereas we did not find any direct correlation between the number of H–bond acceptors and either hydrate propensity or the stoichiometry of the resulting hydrates, analysis of FIMs suggested that hydrates tend to form when the corresponding anhydrate structure does not facilitate intermolecular interactions.

## Abstract 2

To be a hydrate, or not to be a hydrate? That is the question addressed in this study of molecules that are deficient in hydrogen-bond acceptors. The results, although generally inconclusive, suggest that hydrates have a tendency to form when the corresponding anhydrate structure does not facilitate strong intermolecular interactions.

## 1 Introduction

Although crystal engineering^1−4^ has advanced since its inception,^1,2^ there are phenomena that remain poorly understood, including the relationship between the hydrate and anhydrate crystal forms of molecular compounds.^5−9^ Such hydrates are of particular relevance to pharmaceutical science^10−13^ and are therefore a topical subject. The term “hydrate” dates back at least to the early 18th century, when Proust coined it to refer to a “compound of water and other chemicals”.^14^ Hydrate thereby encompasses water adducts of organic molecules (e.g., chloral hydrate), aqua complexes, and crystalline solids that contain water molecules within their crystal lattice.^15,16^ Extensive use of X-ray crystallography enabled visualization of crystal structures and, at least for stoichiometric hydrates, determination of the specific location of water in the crystal lattice. Hydrate as used herein refers to crystalline solids in which water molecules are present within the crystal lattice, including those “heterosolvates”, in which water and a solvent molecule(s) are both present.^17^ On the basis of their experimentally determined crystal structure, molecular hydrates and solvates can be defined as being stoichiometric or nonstoichiometric.^18,19^ Stoichiometric and nonstoichiometric hydrates can be categorized by the ratio of water molecules to organic molecules. Nonstoichiometric hydrates are often referred to as variable hydrates.^20^ In this type of hydrate, the water content varies as a function of the external atmospheric vapor pressure. Morris and Rodriguez-Hornedo defined molecular hydrates as belonging to one of three categories, based on the local environment of water molecules: isolated site hydrates, channel hydrates, and metal-ion-associated hydrates.^21,22^

Isolated site hydrates exhibit structures in which water molecules do not interact with other water molecules, usually because they are engaged in noncovalent interactions with the molecular compound of interest and, therefore, do not form hydrogen bonds with other water molecules. Isolated site hydrates are often stoichiometric. Such hydrates are typically characterized by sharp dehydration endotherms in differential scanning calorimetry (DSC) tests, small weight loss ranges in thermogravimetric analysis until the onset temperature, and sharp hydroxyl bands in infrared (IR) spectroscopy.^21,23^ In channel hydrates, water molecules interact with each other, normally in one-dimensional (1D) channels or two-dimensional (2D) planes. Channel hydrates can be stoichiometric and/or nonstoichiomtric, depending on the size of channel and the extent of hydrogen bonding.^22^ Stoichiometric channel hydrates contain a fixed ratio of water to host compound whereas for nonstoichiomtric channel hydrates water content can change with temperature and humidity. Dehydration of channel hydrates usually occurs at lower temperature, compared to isolated site hydrates,^5^ and may lead to sample amorphization.^24^ In other cases, crystallinity can be preserved after water loss even if significant structural changes result from the collapse of water channels (an effect that can be reversed by exposure of crystals to humidity).^25^ When water molecules do not perform a major structure-sustaining role, the crystal structure can remain intact, with barely any structural alterations.^26−28^ In metal-ion-associated hydrates, water molecules form strong interactions with transition metals or alkali metals.^22^ The resulting hydrated crystal form can show high stability against dehydration.^29^ However, note that the dehydration temperature in metal-ion-associated hydrates will be dependent on the strength of water association and there are cases where water loss is observed to occur below the boiling point of water.^29^ As suggested from above, the dehydration of crystals can differ in its mechanism. There are several studies aimed at understanding this process. Galwey developed a classification for solid-state dehydration.^30^ Concurrently, Petit and Coquerel developed a unified model describing possible mechanisms for the dehydration of molecular crystals,^31^ proposing topological, energetic, and physical criteria that must be considered during the dehydration process.^31^

Hydrates are of technological relevance. Gas hydrates are of interest from the energy and environmental perspectives as they are used for storage and transportation of natural gases and hydrogen.^32,33^ Hydrates can also offer different physicochemical properties, compared to the corresponding anhydrates, thereby affecting materials applications. Recently, an interesting example the effect of water being incorporated into a crystal structure was reported by Zhou et al. In their work, molecular packing was altered by the presence of water, leading to luminogen formation.^34^ The alternative physicochemical properties offered by hydrates are also of interest to the pharmaceutical industry, especially since the presence of water does not raise any serious regulatory concerns as there are no toxicology risks. Indeed, hydrates of active pharmaceutical ingredients (APIs) can ultimately be more suited for use in a drug product than an anhydrate,^21^ as shown by the significant number of drug products formulated as hydrates, e.g., creatine phosphate sodium,^35^ morphine sulfate,^36^ azithromycin,^37^ erythromycin,^38^ and many others.^39−49^ Excipients used in drug products can also be affected by hydrate formation, with lactose, glucose, magnesium stearate and calcium phosphate being examples studied in the literature.^11,50^ However, it is also worth noting that properties of hydrates might be undesirable from the commercial and manufacturing point of view, and their formation can be spontaneous and unintentional.^20,39^ Therefore, control over hydrate formation in molecular compounds is of particular interest to pharmaceutical science, where one-third of drug substances are thought to form crystalline hydrates.^10,20^ Indeed, 31.9% of entries in the European Pharmacopeia (1991) are hydrates and 11.2% are solvates.^51^ Similar statistics for organic compounds were reported from two separate studies in 1999 and 2004 indicating that hydrates are more prevalent (33%) than solvates (10%)^19^ and hydrate formation for organic compounds occurs more frequently than solvate formation with organic solvents.^52^ The relevance of hydrates to pharmaceutical science is discussed in more detail in the Supporting Information.

From a crystal engineering perspective, the propensity of a given molecular compound for hydrate formation, and whether or not such formation is expected, has been a matter of interest for decades.^51,53−55^ 2566 crystalline hydrates retrieved from the Cambridge Scientific Database (CSD) were analyzed by Desiraju et al., and they linked propensity for hydrate formation to an imbalance in the ratio of hydrogen-bond donors and acceptors.^8^ Infantes et al. conducted a study based on 3258 hydrate crystal structures retrieved from the CSD and concluded that hydrate formation does not correlate with the hydrogen-bond donor/acceptor ratio. Rather, they identified the sum and/or difference in the total number of hydrogen-bond donors and acceptors, molecular polarity, and the presence of charged atoms or groups as factors that impact hydrate formation.^9,56,57^ Moreover, it has been reported that the donor/acceptor ratio affects the hydrogen bond pattern of water in hydrates. An excess of donors favors patterns where water molecules act as H-atom acceptors, while donor deficiency leads to patterns where water molecules serve H-atom donors.^9^ Note that both studies were based on statistical analysis of crystal structures archived in the CSD. While the CSD is a gold mine of organic and metal–organic structural data with over 1 million deposits,^58^ it is likely that it underestimates hydrate occurrence^7^ and the number of compounds for which both hydrate and anhydrate crystal forms are known is relatively low. In a recent study by Werner and Swift a search method was developed describing how CSD Python API based on Simplified Molecular Input Line Entry Strings (SMILES) enables searching for molecular hydrates and their corresponding anhydrate crystal structure in CSD.^59^ The results of this study revealed that, whereas more than 23 000 molecular hydrates (with no metal ions) were retrieved from the CSD, only ∼2000 hydrate–anhydrate pairs were found.^59^ They also reported that a significant number of hydrates in hydrate–anhydrate pairs have a tendency to crystallize in a lower symmetry compared to their anhydrous form.^59^ In order to address the propensity of an organic compound to crystallize as a hydrate, the statistical frequency of the occurrence of crystalline hydrates,^9^ conditions for their formation,^9,11,21^ and preferred chemical environments for water molecules,^57,60,61^ have been investigated. We recently conducted a systematic study of molecular compounds that aimed to address the following questions:^7^

(i) Can the tendency of a given organic compound to form hydrate(s) be predetermined?
(ii) How common is hydrate formation for molecular organic compounds?
(iii) What are the most effective experimental methods to discover hydrates?

**Scheme 1:**
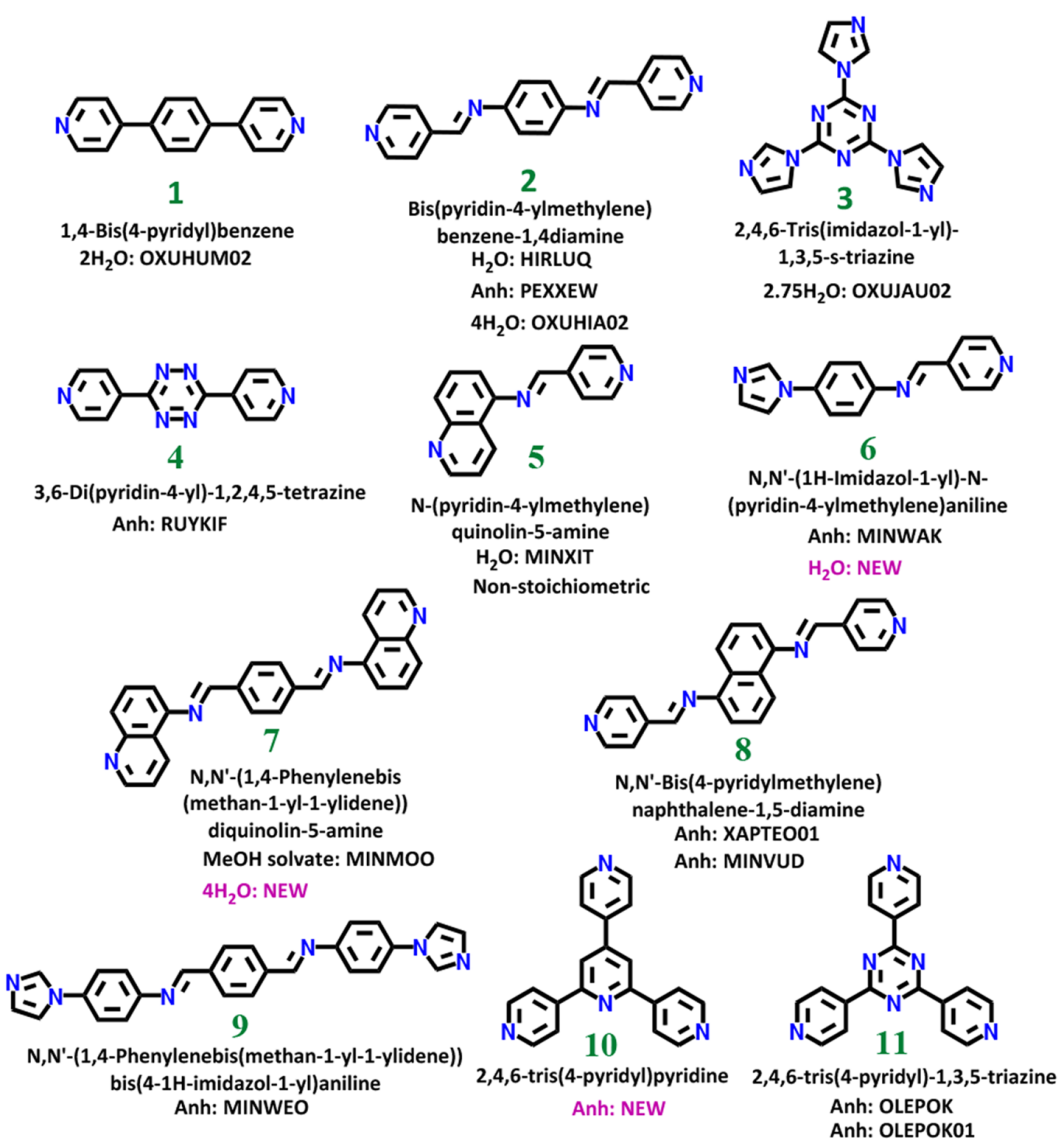
Library of *N*-Heterocyclic Compounds Investigated Herein for Their Propensity to Form Hydrates REFCODEs for structures reported in the CSD of anhydrate (Anh) and hydrate (H_2_O) forms, and previously unreported structures (New) are listed.

The previous study by our group highlighted how systematic hydrate screening experiments,^62−65^ in conjunction with statistical CSD analyses, can provide insight into the propensity of a molecular compound for hydrate formation.^7^ The screening experiments revealed that the hydrate propensity of a family of molecular compounds that lack strong hydrogen-bond donors was much greater than expected from CSD statistics (73% vs 18%). However, note that the values resulting from CSD statistics can be artificially understated by the small number of systematic studies aimed at hydrate formation. Furthermore, the role of crystal packing in anhydrates was determined to be a factor that governs hydrate propensity.^7^ Herein, we address three additional questions through expanding our library of molecular compounds: (i) What happens if the number of hydrogen-bond acceptors is increased? (ii) Does the number of hydrogen-bond acceptors correlate to the stoichiometry of water in the crystal lattice? (iii) How important is the role of crystal packing in comparison to the lack of strong hydrogen-bond donors? Three compounds (**1**–**3**; see Scheme 1) in our previous study, readily yielded hydrates from hydrate screening experiments.^7^ Variants of **1**–**3** were selected and subjected to systematic hydrate screening^62−65^ using the following five approaches: (i) recrystallization from mixed solvent systems; (ii) slurry in water at ambient temperature; (iii) exposure of dry powders to humid conditions; (iv) aqueous solvent drop grinding (SDG); and (v) dynamic water vapor sorption (DVS).

## 2 Experimental Section

### 2.1 General Aspects

All reagents and solvents were purchased from Alfa Aesar, AK Scientific, or Sigma–Aldrich and were used as received. Powder X-ray diffraction (PXRD) data were collected on a Philips X’Pert PRO MPD equipped with a Cu Kα source. Data were collected from 5° to 40° 2θ, using a step size of 0.02° at a scan rate of 0.1° min^–1^. Thermogravimetric analyses (TGA) were measured on a TA Instruments Q50 TG from room temperature (rt) to 500 °C at a scan rate of 20 °C min^–1^, under a 60 mL min^–1^ flow of N_2_. Dynamic vapor sorption (DVS) measurements were performed at 25 °C on a Surface Measurement Systems DVS Intrinsic instrument, using air as a carrier gas to gravimetrically measure the uptake and loss of vapor. The mass of the sample was determined by comparison to an empty reference pan and recorded by a high-resolution microbalance with a precision of 0.1 μg. Sorption isotherms were measured stepwise between 0 and 95% relative humidity (RH) with a convergence equilibrium criterion d*m*/d*t* = 0.01 % min^–1^.

### 2.2 Single-Crystal X-ray Crystallography of **7·**4H_2_O and **10**

Single-crystal X-ray diffraction (SCXRD) data were collected on a Bruker D8 Quest diffractometer equipped with a μS microfocus Cu anode (λ = 1.54178 Å) and Photon II detector. For low-temperature measurements, an open-flow nitrogen attachment from Oxford Cryosystems was used. Data were indexed, integrated, and scaled with APEX3.^66^ Absorption corrections were performed by a multiscan method using SADABS.^67^ Space groups were determined using XPREP^68^ implemented in APEX3. The SHELX-2014 program package, implemented in OLEX2 v1.2.8,^69^ was used for structure solution and refinement. Structures were solved using an intrinsic phasing method (SHELXT)^70^ and refined with SHELXL^71^ using a least-squares method. All non-hydrogen atoms were refined anisotropically. Hydrogen atoms were fixed from the molecular geometry at idealized positions and assigned isotropic thermal parameters based on the equivalent displacement parameters of the atoms to which they are binded. Crystallographic data were deposited with the Cambridge Crystallographic Data Centre (CCDC, File Nos. 1859837 and 1859838). Crystallographic data and refinement parameters for all crystals structures are given in Table S1a in the Supporting Information (SI).

### 2.3 Crystal Structure of **6·**H_2_O from PXRD Data

The program DASH^72^ was used for lattice parameters and space group determination (using the Pawley method), structure solution (using the simulated annealing method), and preliminary Rietveld refinement. The final Rietveld refinement was performed using the program GSAS-II.^73^ All atoms were refined isotropically. Hydrogen atoms were placed from the molecular geometry at idealized positions. Crystal structure data has been deposited with the CCDC (CCDC 1897070). Crystallographic data and refinement parameters are reported in Table S1b in the SI and comparative patterns for the observed and calculated intensities including their difference are shown in Figure S19 in the SI.

### 2.4 Syntheses of Compounds **1**–**11**

**1**–**11** were prepared following procedures reported in the literature.^74−77^**5**–**9** were prepared by a facile synthetic strategy, co-crystal controlled solid-state synthesis, developed by us.^77^ Single crystals of **7·**4H_2_O and **10** were isolated by slow evaporation of a saturated solution of either **7** or **10** in CHCl_3_/MeOH (1:1 v/v) over 5 days. **7·**4H_2_O (97% yield) and **10** (56% yield) were isolated as needle-shaped crystals. Detailed accounts of the syntheses of compounds **1**–**11** are provided in the SI.

### 2.5 Slurry Experiments

50 mg of each compound was slurried for up to 7 d in a sealed glass vial at room temperature in a solvent system (H_2_O or EtOH/H_2_O) acceptable for use in the pharmaceutical industry. The volume of solvent used to suspend the sample was one-third of the volume required to dissolve it completely. Aliquots were removed in order to collect PXRD and TGA data. Details of the slurry experiments for **4**–**11** are provided in the SI.

### 2.6 Stability Tests

The anhydrate forms of **4**–**11** were subjected to stability testing by placing 50 mg of each compound in a humidity chamber under 75% RH at 40 °C. Aliquots were removed after 7 and 14 d and PXRD data were collected.

### 2.7 Solvent Drop Grinding (SDG) Experiments

20 mg of each anhydrate and 10 μL of water were manually ground using an agate mortar and pestle until the initial paste became a fine powder (ca. 10 min). The resulting solid was characterized by PXRD and TGA.

### 2.8 Dynamic Vapor Sorption (DVS) Experiments

DVS measurements were performed on ca. 20 mg of each anhydrate at 1 atm using a DVS Intrinsic instrument. After the experiment, each sample was characterized by PXRD and TGA.

### 2.9 Electrostatic Potential (ESP) Map Calculations

The atomic positions of **4**–**11** were optimized using density functional theory (DFT) with the 6-31G* basis set applied to all atoms and the M06-L hybrid functional.^78^ The optimization calculations were performed using the NWChem ab initio simulation software^79^ on the Comet supercomputer (XSEDE/San Diego Supercomputer Center). For each molecule, a three-dimensional (3D) surface around the molecule was calculated in which the electron density was equal to 0.002 a.u. The resulting isodensity surface served as the basis for mapping the electrostatic potential. The electrostatic potentials of **4**–**11** were then calculated using the same level of theory. A graphical representation of the electrostatic potential surface for **4**–**11** was generated using Spartan’16 software.^80^

### 2.10 Full Interaction Map (FIM) Calculations

Full interaction maps (FIMs) presented in this study were calculated using the program Mercury.^81^ In order to show the possible interaction landscape only when molecules of water and **4**–**11** are present in the crystal structure, two probes, water oxygen and aromatic C–H carbon functional groups, were selected. For crystal structures with more than one symmetrically independent N-heterocyclic molecule per asymmetric unit, FIMs were calculated and presented for each conformationally unique molecule.

## 3 Results and Discussion

We previously suggested that dihydrates had a tendency to occur for linear diaza compounds (dipyridyls), where the water molecules can form C2 chains.^7^ We also observed that trihydrates and tetrahydrates can occur for trisubstituted *N*-heterocyclic aromatics and imines, respectively. In order to further address hydrate propensity and if there is a correlation between the number of hydrogen-bond acceptors and the stoichiometry of water molecules in hydrates, we selected eight molecules containing five- and six-membered *N*-heterocyclic aromatic functional groups that are rich in *N*-donors (Scheme 1). These compounds (classified as relatively hydrophobic) can be subdivided as follows: class (i) linear diaza compounds (**1** and **4**); class (ii) imines with conformational flexibility (**2** and **5**–**9**), and class (iii) trisubstituted *N*-heterocyclic aromatics (**3**, **10**, and **11**).

### 3.1 Hydrate Screening Experiments

**4**–**11** were screened for their propensity toward hydrate formation to follow from our study of **1**–**3**. **1** and **4** are linear molecules of the type that can serve as linker ligands in coordination polymers, whereas **2** and **5**–**9** are able to exhibit torsional flexibility and remain underexplored as ligands. Trisimidazolyltriazine **3** and trispyridyls **10** and **11** have limited torsional flexibility. The hydrate screening^62−65^ experiments conducted were as follows: (i) crystallization from mixed solvent systems; (ii) slurry in water at ambient temperature; (iii) exposure of dry powders to humid conditions; (iv) aqueous solvent drop grinding (SDG); and (v) dynamic water vapor sorption (DVS). The results of these hydrate screening experiments are summarized in Table 1.

**Table 1:** Results of Hydrate Screening Experiments^a^

compound	slurry in H_2_O	75% RH/40 °C	DVS	SDG
**1**	H	H	H	H
**2**	H	H	A	H
**3**	H	H	H	H
**4**	A	A	A	A
**5**	H	H	H	H
**6**	H	H	H	H
**7**	H	H	H	H
**8**	A	A	A	A
**9**	A	A	A	A
**10**	A	A	A	A
**11**	A	A	A	A

a A = anhydrate, H = hydrate, DVS = dynamic water vapor sorption, and SDG = solvent drop grinding.

Crystallization from mixed-solvent systems afforded two new crystal structures: a hydrate (**7**) and an anhydrate (**10**) (see Scheme 1). Water slurries were performed on dried samples of **4**–**11** under ambient conditions. Of the eight compounds studied, three (**5**–**7**) yielded hydrates. The thermal stability of hydrates (**5**–**7**) was evaluated by TGA (see the SI). TGA of the hydrates of **5** and **7** revealed water loss below 100 °C. However, loss of water molecules below 100 °C can also occur for isolated-site hydrates, such as the monohydrates of **6** and **2** (HIRLUQ). To further examine the relative stability of the isolated hydrates, competitive slurry experiments in EtOH were conducted. The anhydrate and hydrate forms (1:1 w/w ratio) of **5**–**7** were slurried in various ratios of EtOH and H_2_O (see the SI for full details). Whereas the hydrate forms of **5** and **6** were isolated, even with a 5:1 EtOH/H_2_O ratio, **7** required a 1:1 or lower ratio of EtOH/H_2_O (SI). Hydration experiments involving slurrying in water or water/organic solvent mixtures revealed changes in the PXRD patterns versus the corresponding anhydrous forms. For humidity tests (method (iii)), standard accelerated stability testing conditions used in pharmaceutical industry (40 °C and 75% RH) were employed.^82^ The anhydrous forms of **4**–**11** were exposed to 75% RH at 40 °C for a minimum of 7 d and hydrate forms of **5**–**7** were then isolated, the level of hydration being confirmed by TGA. Anhydrous forms of **5**–**7** were observed to transform to hydrated forms of **5**–**7** following aqueous slurry and exposure to humidity. Anhydrous samples of **4**–**11** were also subjected to aqueous SDG.^77,83,84^ Hydrated forms of **5**–**7** were isolated from the SDG experiments.

The results of DVS testing are presented in Figure S1 in the SI. DVS measurements on anhydrous forms of **1**–**3** were not presented in our previous study, therefore, these results are also included herein. Compound **1** is known to form a dihydrate.^7^ The adsorption isotherm of **1** revealed an uptake of 11.7 wt % at 95% RH, corresponding to 1.5 water molecules per molecule of **1**. The anhydrous form of **3** exhibited an uptake of 18.7 wt % at 95% RH, which corresponds to ∼2.8 water molecules per molecule of **3**. This result is consistent with the previously reported crystal structure of the hydrate of **3**, where the asymmetric unit is comprised of 5.5 molecules of water and two molecules of **3**, corresponding to a 2.75:1 water:**3** ratio. The desorption cycle exhibited strong hysteresis indicating that the hydrated form of **3** is stable at as low as 20% RH. **5** also formed a hydrate during DVS as revealed by an uptake of 11.1 wt % at 95% RH, corresponding to an H_2_O:**5** ratio of 1.4:1. The desorption cycle exhibited strong hysteresis indicating that the hydrated form of **5** is stable to as low as 15% RH. The anhydrous form of **6** exhibited an uptake of 7.4 wt % at 95% RH, which corresponds to a monohydrate. The anhydrous form of **7** exhibited a water uptake of 5.8 wt % at 95% RH, which also corresponds to a monohydrate. The desorption cycle of **7** exhibited hysteresis, indicating that the hydrated form of **7** is stable down to conditions as low as 25% RH. The anhydrous forms of **2**, **4**, **8**, **9**, **10**, and **11** were found to exhibit water uptake consistent with surface moisture.

Overall, the hydrate screening experiments above revealed that only 3 out of the 8 molecular compounds studied herein (37.5%, **5**-**7** in Scheme 1) formed hydrates. This compares to 8 of the 11 (72.7%) molecular compounds studied in our previous report.^7^ Slurrying of anhydrous samples in water under ambient conditions (method (ii), exposure of dry powders in a humidity chamber (method (iii) and aqueous SDG (method (iv) proved to be the most effective methods to generate hydrates. The inconsistency in hydrate formation via DVS in comparison to the other techniques suggests that it is important to consider more than one method in assessing hydrate propensity. It should be noted that any anhydrate–hydrate transformation that requires crystal packing modification could mean slow kinetics and DVS (not exceeding 24 h) is therefore less likely to induce transformation than long-term humidity experiments (7 or 14 days). For slurry experiments, transformation might occur more readily because dissolution and recrystallization is possible. We also note that our DVS tests were performed at 25 °C vs 40 °C for the humidity chamber tests. The higher water content in the humidity chamber could increase the rate of transformation. Molecular rearrangement is further discussed in section C.

### 3.2 Computational Studies

**Figure 1:**
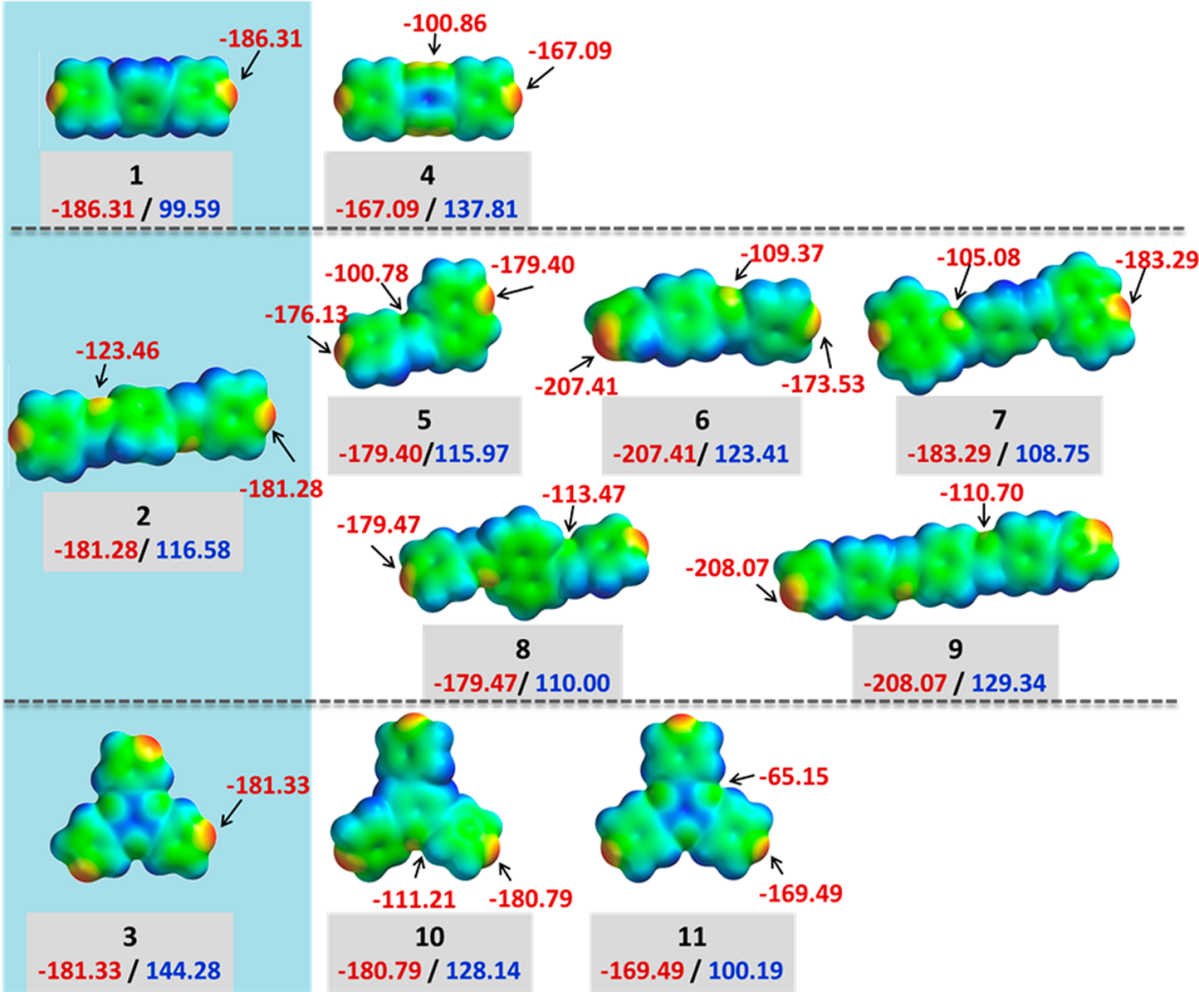
Electrostatic potential maps (kJ mol^–1^) for **1**–**11**.^80^

The electrostatic potential (ESP) of a molecule in its gas phase can provide insight into the following matters: (i) intermolecular interactions and crystallization behavior;^85−87^ (ii) the ability of a molecule to accept/donate a proton in solution;^88,89^ and (iii) hydrogen-bonding energies.^90^ In order to evaluate if the ESP at the N atoms correlates with hydrate propensity, electrostatic potentials were calculated for **4**–**11** and mapped on the molecular electron density surfaces (Figure 1).

**4** was not observed to form a hydrate and has a much lower negative potential (−167.09 kJ mol^–1^) than **1**, which readily formed a dihydrate (−186.31 kJ mol^–1^). The relatively weak negative electrostatic potential implies that **4** would not be as strong a hydrogen-bond acceptor from water. For **5**-**9**, the negative potentials on the aromatic N atoms range from −173.53 to −208.07 kJmol^–1^. Interestingly, **8** and **9**, despite negative potentials of −179.47 and −208.07 kJmol^–1^, respectively, failed to form hydrates during our screening experiments, while **5** (negative potential of −179.4 kJmol^–1^) readily formed a hydrate. **3** has a negative potential of −181.33 kJ mol^–1^ and readily formed a trihydrate. Meanwhile, neither **10** nor **11** (with negative potentials of −180.79 and −169.49 kJ mol^–1^, respectively) formed hydrates. The large negative potentials on the aromatic N atoms of **8**–**10** would suggest a strong propensity to form hydrates. The fact that **8**–**10** did not form hydrates indicates that other factors, such as the role of intermolecular interactions in crystal packing could be a factor as discussed below. The findings from ESP maps and comparison of the negative potential values of structurally similar molecules suggests that consideration of only the electrostatic potential is not necessarily sufficient for prediction of hydrate formation.

### 3.3 Crystal Packing Analysis

**Figure 2:**
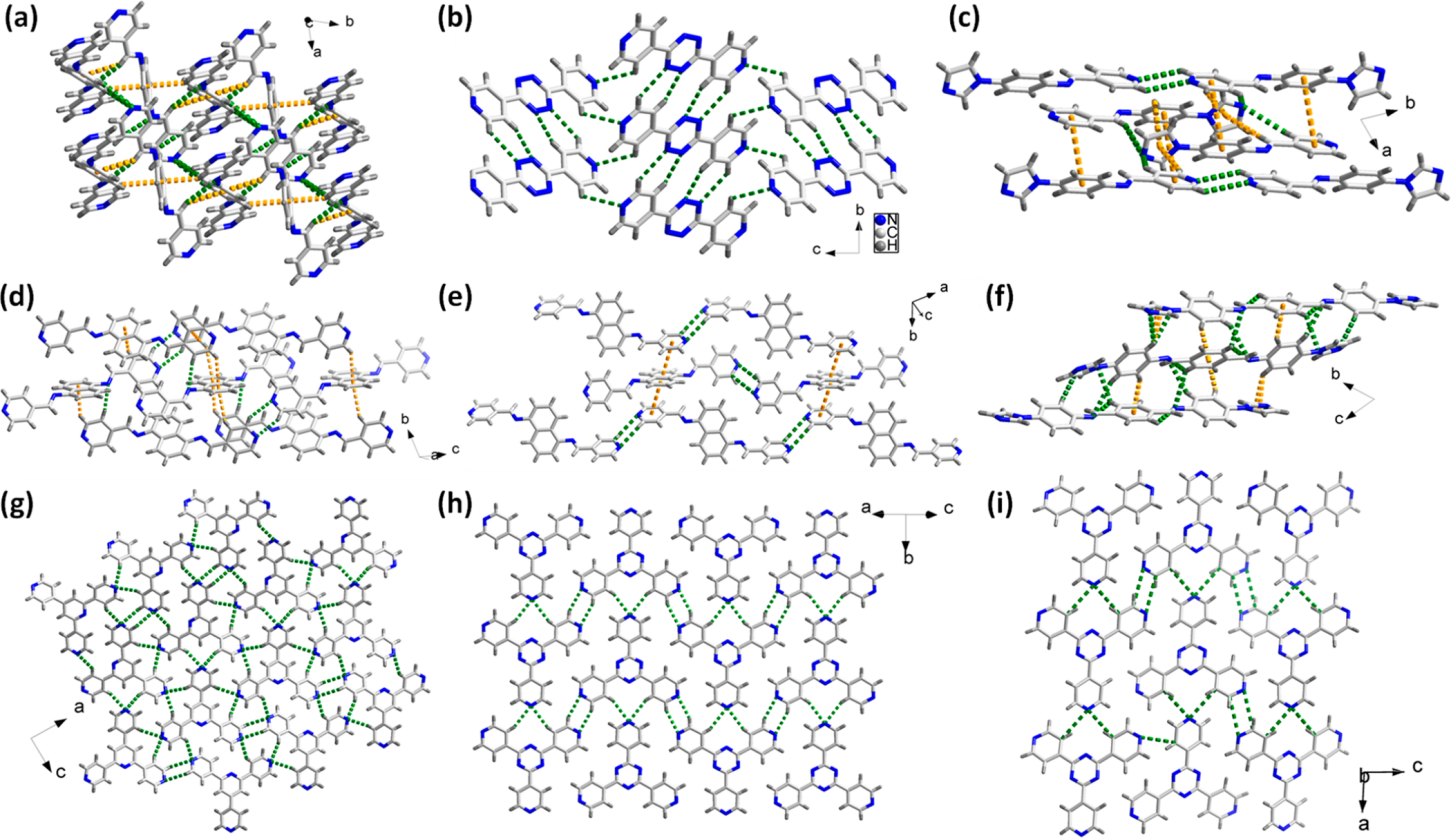
Crystal structures depicting multiple intermolecular interactions (C–H**···**N shown in green; C–H**···**π and π–π shown in yellow) in the anhydrates of (a) **2** (PEXXEW), (b) **4** (RUYKIF), (c) **6** (MINWAK), (d) **8** (XAPTEO01), (e) **8** (MINVUD), (f) **9** (MINWEO), (g) **10**, (h) **11** (OLEPOK) and (i) **11** (OLEPOK01).

**Figure 3:**
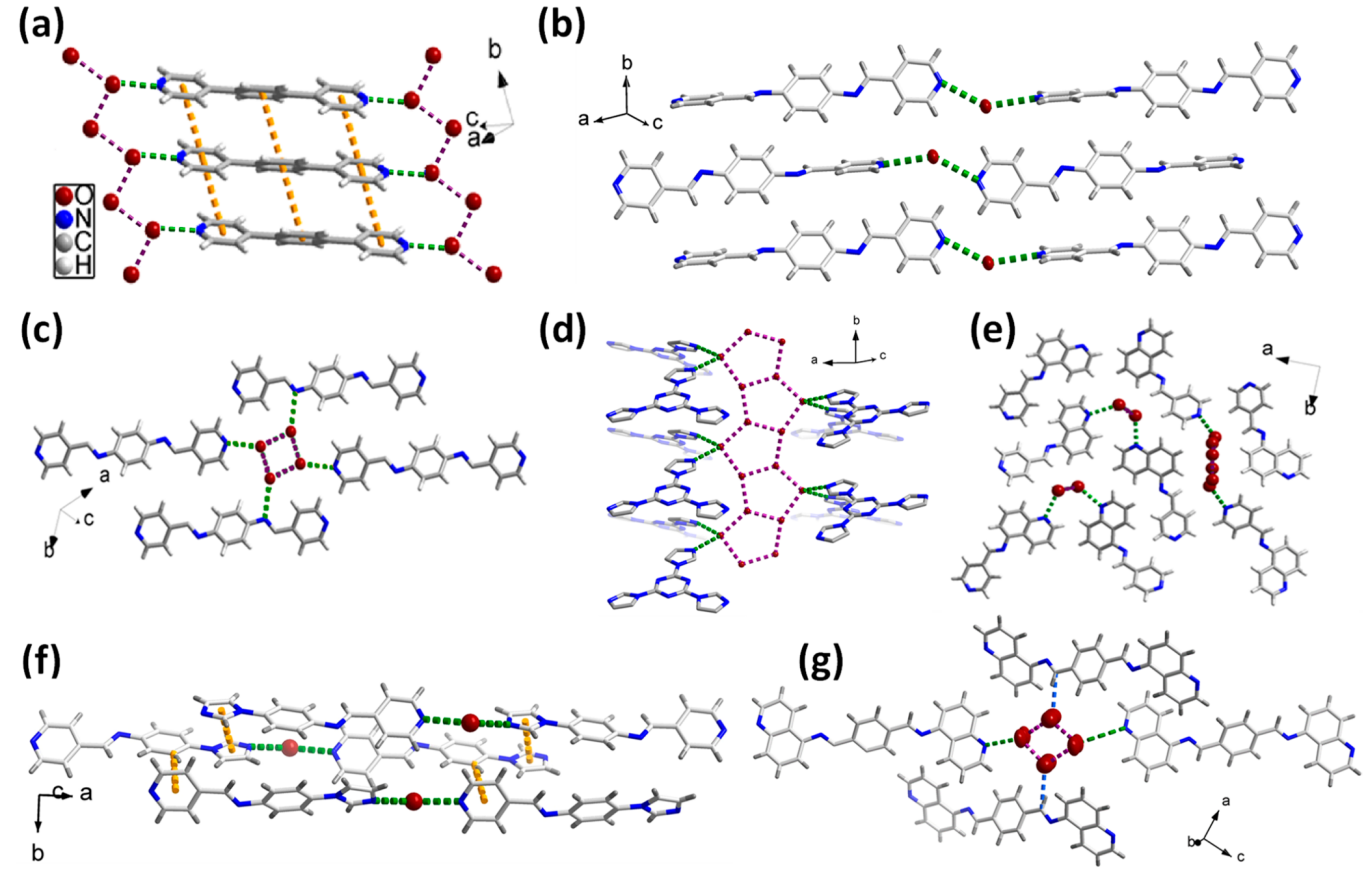
Crystal structures depicting selected intermolecular interactions (O–H**···**N shown in green; π–π shown in yellow; O–H**···**O shown in purple; C–H**···**O shown in blue) in the hydrates of (a) **1·**2H_2_O (OXUHUM02), (b) **2·**H_2_O (HIRLUQ), (c) **2·**4H_2_O (OXUHIA02), (d) **3·**3H_2_O (OXUJAU02), (e) **5·***x*H_2_O (MINXIT), (f) **6·**H_2_O, and (g) **7** 4H_2_O. Hydrogen atoms of water molecules were omitted for the sake of clarity.

The crystal packing motifs of the anhydrate and hydrate forms of **1**–**11** were analyzed. Figures 2 and 3 highlight the intermolecular interactions that impact crystal packing. In the crystal structures of the anhydrates, multiple weak C–H**···**π and/or C–H**···**N interactions control crystal packing (Table S2 in the SI). In the crystal structures of the hydrates, the crystal packing has a tendency to be directed by O–H**···**N hydrogen bonds between water molecules and N atoms and O–H**···**O hydrogen bonds between water molecules. In our previous study, we observed that, for dihydrates and trihydrates with 1D water motifs, water aggregation occurs in such a manner to enable π–π stacking of the organic molecules.^7^ As a result, the crystal structures of such hydrates are sustained not only by strong O–H**···**O and/or O–H**···**N hydrogen bonds, but also by π–π stacking interactions (Figures 3a and 3d). This type of crystal packing was also observed herein (Figure 3f). While compound **1** exemplifies a hydrated crystal structure sustained by all three types of intermolecular interactions mentioned above, **4** failed to form a hydrate in our screening experiments. As mentioned in section 3.2, the electrostatic potential of N atoms in compound **4** might reduce its propensity for hydrate formation. Additionally, crystal packing in the reported anhydrous form of **4** is directed by C–H**···**N and face-to-face π–π stacking interactions (Table S2 in the SI), which may be a factor working against hydrate formation in a manner similar to observations from our previous study.^7^**2** is known to exist as both monohydrate and tetrahydrate forms. The monohydrate form of **2** is an isolated site hydrate where water molecules form O–H**···**N and C–H**···**O hydrogen bonds with molecules of **2** (Table S2). The crystal structure is further sustained by C–H**···**N as well as C–H**···**π interactions. In the tetrahydrate form, water molecules form tetramers that are hydrogen-bonded to molecules of **2** (Figure 3c) to generate sheets. The mode of packing and conformation of molecules of **2** limit π–π stacking interactions with only one such example at a distance of 4.1 Å. Consideration of only the electrostatic potential and intermolecular interactions present in crystal structures are not necessarily sufficient to address flexible molecules as exemplified by **5**-**7**. An important consideration for **5** and **7** is their bend, which hinders the type of dense packing possible in the anhydrate forms of linear molecules such as **6**, **8**, and **9**.^91,92^ It was previously shown that when the shape of a molecule precludes dense packing, the presence of solvent or water molecules in solvates or hydrates, respectively, might be anticipated.^92,93^ The anhydrous form of **6** crystallized readily, yielding single crystals suitable for SCXRD.^77^ The hydrate of **6** also forms readily, however, attempts to isolate single crystals were unsuccessful. Therefore, the crystal structure of the hydrated form of **6** was determined from PXRD data (see Table S1b and Figure S18 in the SI).

The anhydrate form of **6** was found to exhibit crystal packing governed by multiple weak C–H**···**N and C–H**···**π interactions (see Table S2 and Figure S18a in the SI). Molecules of **6** are arranged in a tail-to-tail fashion through C–H**···**N interactions between pyridyl and imidazolyl rings. In the hydrated form of **6**, the number of intermolecular interactions between molecules of **6** decreases (see Table S2), hydrate formation being supported by O–H**···**N hydrogen bonds between water molecules and N atoms in addition to C–H**···**π and π**···**π interactions (Table S2 and Figure 3f). In the hydrated form of **6**, water molecules are positioned between the pyridyl and imidazolyl moieties to generate hydrogen bonded chains. The resulting head-to-tail arrangement of molecules of **6** in the hydrate form differs from the tail-to-tail aggregation in the anhydrate of **6**.

Investigation of the remaining two molecules in class (ii), compounds **8** and **9**, revealed that the propensity of **9** to exist as an anhydrate might be attributed to the presence of many weak C–H**···**N as well as C–H**···**π interactions that preclude hydrate formation. Each molecule of **9** is involved in 28 C–H**···**π interactions and, depending on their conformation, 18 or 10 C–H**···**N hydrogen bonds (Table S2). In the case of **8**, both anhydrate polymorphs exhibit C–H**···**N and C–H**···**π interactions (Table S2). In addition, the crystal packing in **8** (MINVUD) is directed by several face-to-face π–π stacking interactions (Table S2). The fact that **8** did not form a hydrate can be attributed to the unfavorable electrostatic potential of its N atoms, as discussed in section 3.2.

**3** was observed to form a hydrate but its anhydrous structure remains unknown. **10** and **11** did not form hydrates in our screening experiments. The electrostatic potential of the N atoms in **10** is similar to compound **3**, and its existence in anhydrous form is likely a consequence of the numerous C–H**···**N and π–π stacking interactions that sustain its crystal structure. Each molecule of **10** is involved in 12 C–H**···**N and 8 π–π stacking interactions (see Table S2). In the case of **11**, both anhydrate polymorphs are directed by C–H**···**N and face-to-face π–π stacking interactions (Table S2), which may be a factor working against hydrate formation, similar to observations from our previous study.^7^ For both, OLEPOK and OLEPOK01, each molecule of **11** is involved in 8 C–H**···**N hydrogen bonds, and in 4 and 8 π–π stacking interactions, respectively (Table S2). The unfavorable electrostatic potential of the N atoms in **11** may prelcude hydrate formation.

**Figure 4:**
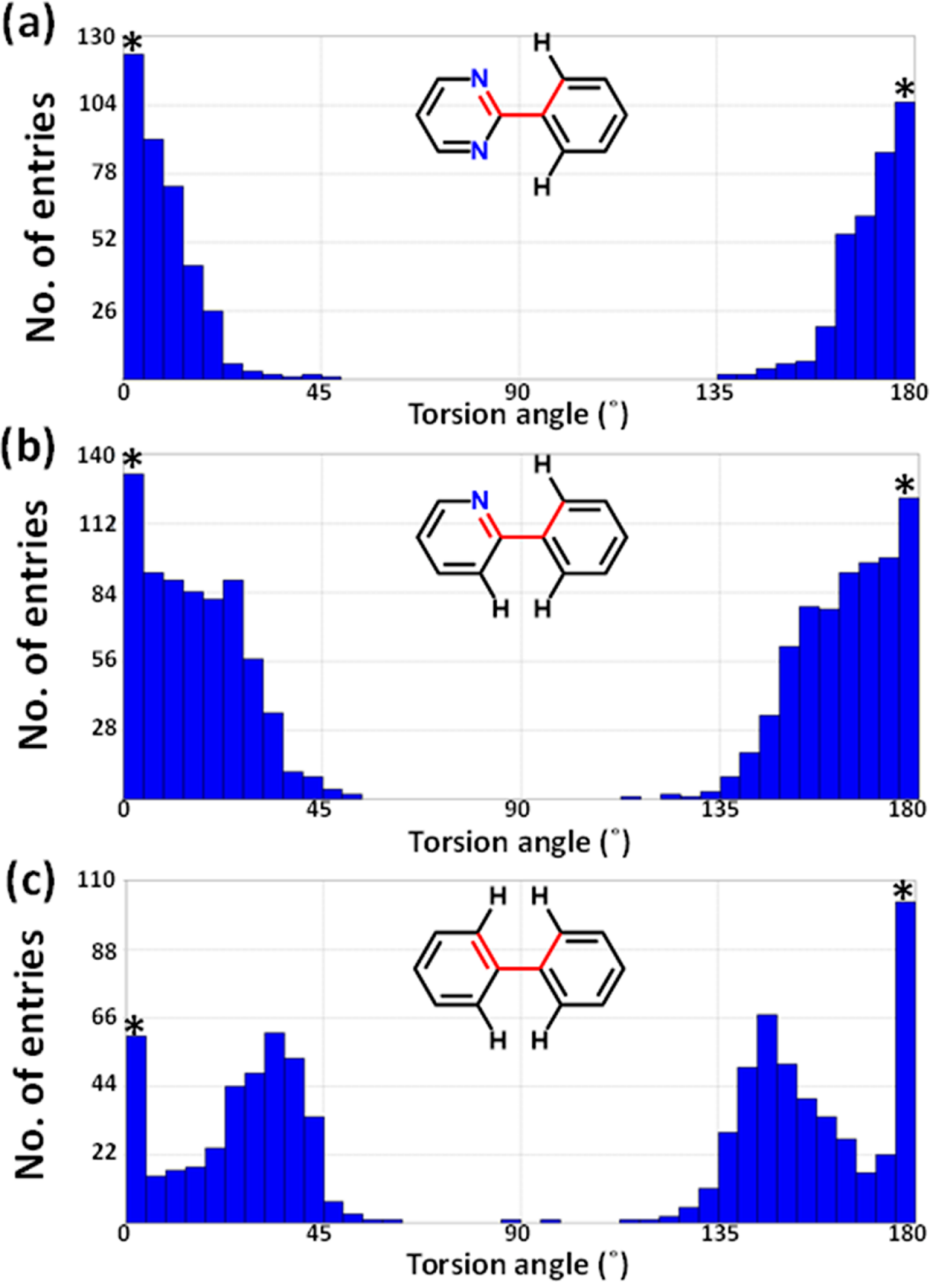
Histograms showing the distribution of dihedral angles about two linked aryl rings in entries archived in the CSD. The number of structures exhibiting a torsion angle close to 0° or 180° (marked with asterisks) can also include structures with molecules on special positions for which disorder by symmetry was not taken into account.

The intermolecular interactions observed in the crystal structures of the hydrates and anhydrates discussed in this section are listed in Table S2. Despite multiple attempts, we were unsuccessful in isolating single crystals of all hydrate–anhydrate pairs. Moreover, our results do not follow the rule of increasing the hydrate propensity when the number of hydrogen-bond acceptors increased. For example, despite an increase in the number of N atoms accessible for hydrogen bonding vs **1**, a hydrate was not formed by **4**. In order to gain insight, a CSD^94^ survey (ConQuest^95^ 2020.3.1, CSD v5.42 November 2020, R factor <5.0%, no organometallics, no powder structures) to examine preferred conformations between aryl rings was conducted. Histograms (Figure 4) revealed that the torsion angle between two linked aryl rings tends toward planarity when N atoms are in the *ortho* position, which is in agreement with the study done by Janczak et al. When only CH moieties are present in the *ortho* positions, steric hindrance tends to result in a nonplanar conformation in the solid state, with a torsion angle ranging from 22 to 40° (Figure 4c). Nevertheless, our analysis has also shown a high number of structures with 0° and 180° torsion angles (Figure 4c), which is consistent with previous study by Brock and Minton, where the occurrence of nearly planar biphenyl fragments was observed to be higher than expected.^96^ When one of the *ortho* positions is replaced with a N atom, the biphenyl moiety is less constrained, with torsion angle ranges from 4° to 35° (Figure 4b). Steric hindrance is further reduced when the second CH moiety is replaced with a N atom. The aryl rings then tend toward planarity, facilitating molecules to pack via π–π stacking interactions. Such interactions can result in high packing density and mitigate against hydrate formation. These observations may explain **4** and **11** (OLEPOK). In **10**, the core pyridyl ring means that there are two types of aryl environment (Figures 4b and 4c). The torsion angles between the aryl rings in *ortho* positions and the pyridyl moiety range from 15.6° to 19.3° while the ring in the *para* position exhibits a torsion angle of 39.1°. In both cases, experimentally observed torsion angles correlate with Figure 4. Despite the nonplanar conformation of molecules in **10**, all aryl rings engage in π–π stacking interactions that could mitigate against the formation of a hydrate.

### 3.4 Full Interaction Map (FIM) Analysis

**Figure 5:**
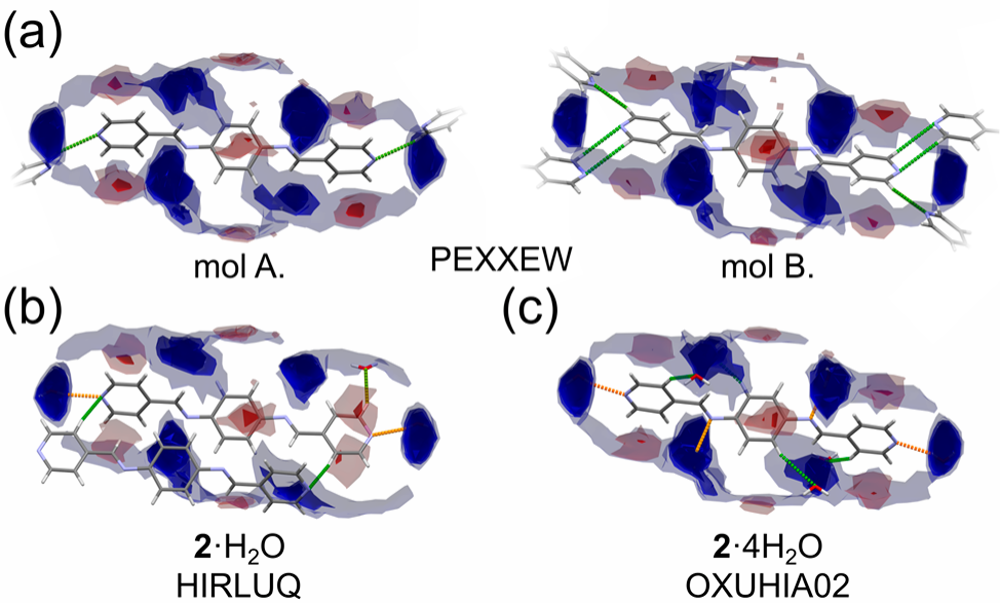
Full interaction maps (FIMs) for the molecular conformers present in crystal structures of **2**: (a) anhydrate (CSD refcode PEXXEW), (b) monohydrate (CSD refcode HIRLUQ), and (c) tetahydrate (CSD refcode OXUHIA02). The blue and red contours indicate regions most commonly taken by hydrogen-bond-involved water molecule and aromatic C–H moieties, respectively. The opacity of the region is positively corelated to the probability of the interaction existence. A color scale (from green, through orange, to red) was applied for hydrogen bonds to mark their relative length (green being the longest and red being the shortest). For an expanded version of the figure, see Figure S20 in the SI.

**Figure 6:**
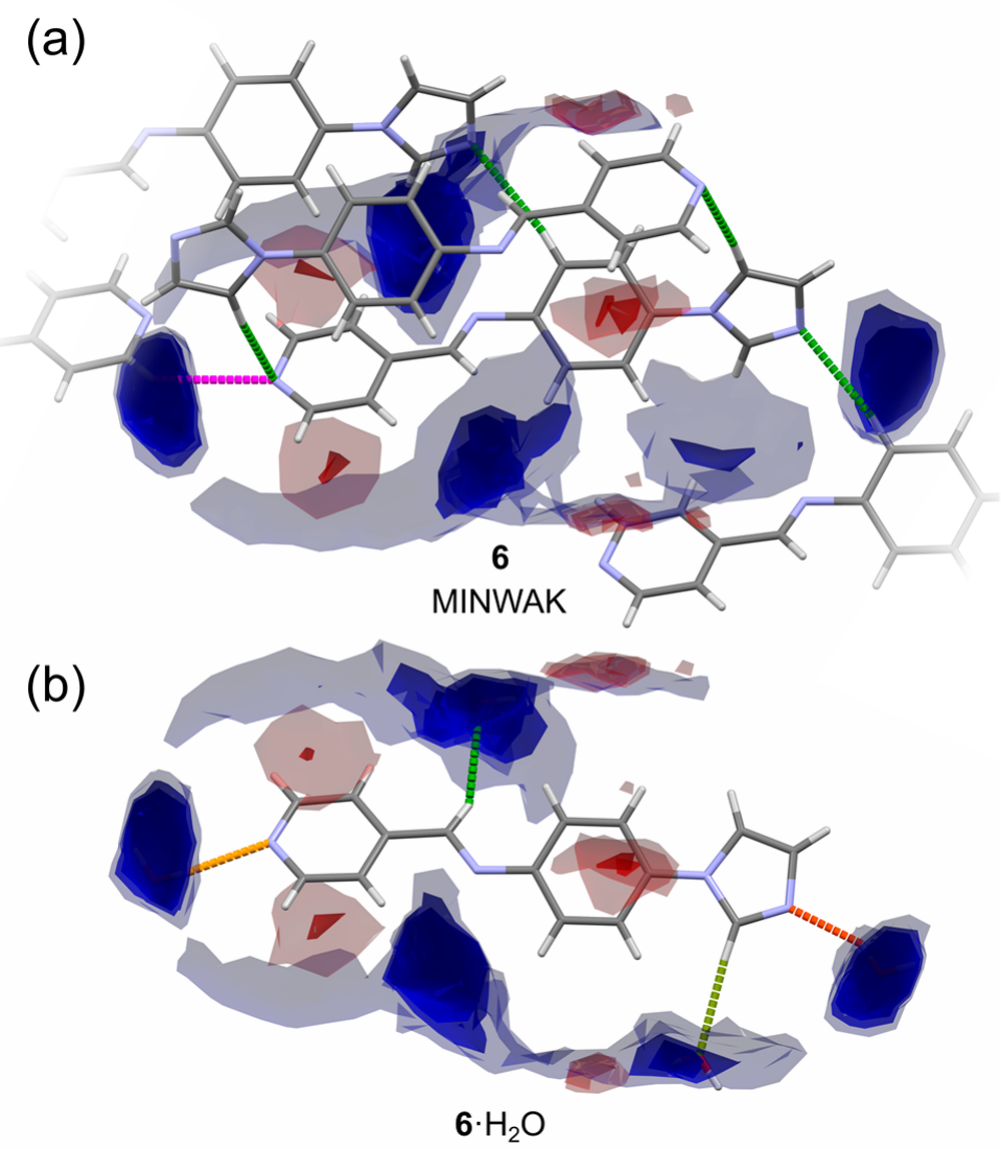
Interaction preferences for the molecular conformation of (a) anhydrate (MINWAK) and (b) hydrate forms of **6**. For a detailed description of the color coding, see the caption for Figure 5. Possible very long contacts are marked in magenta. For an expanded version of the figure, see Figure S22 in the SI.

**Figure 7:**
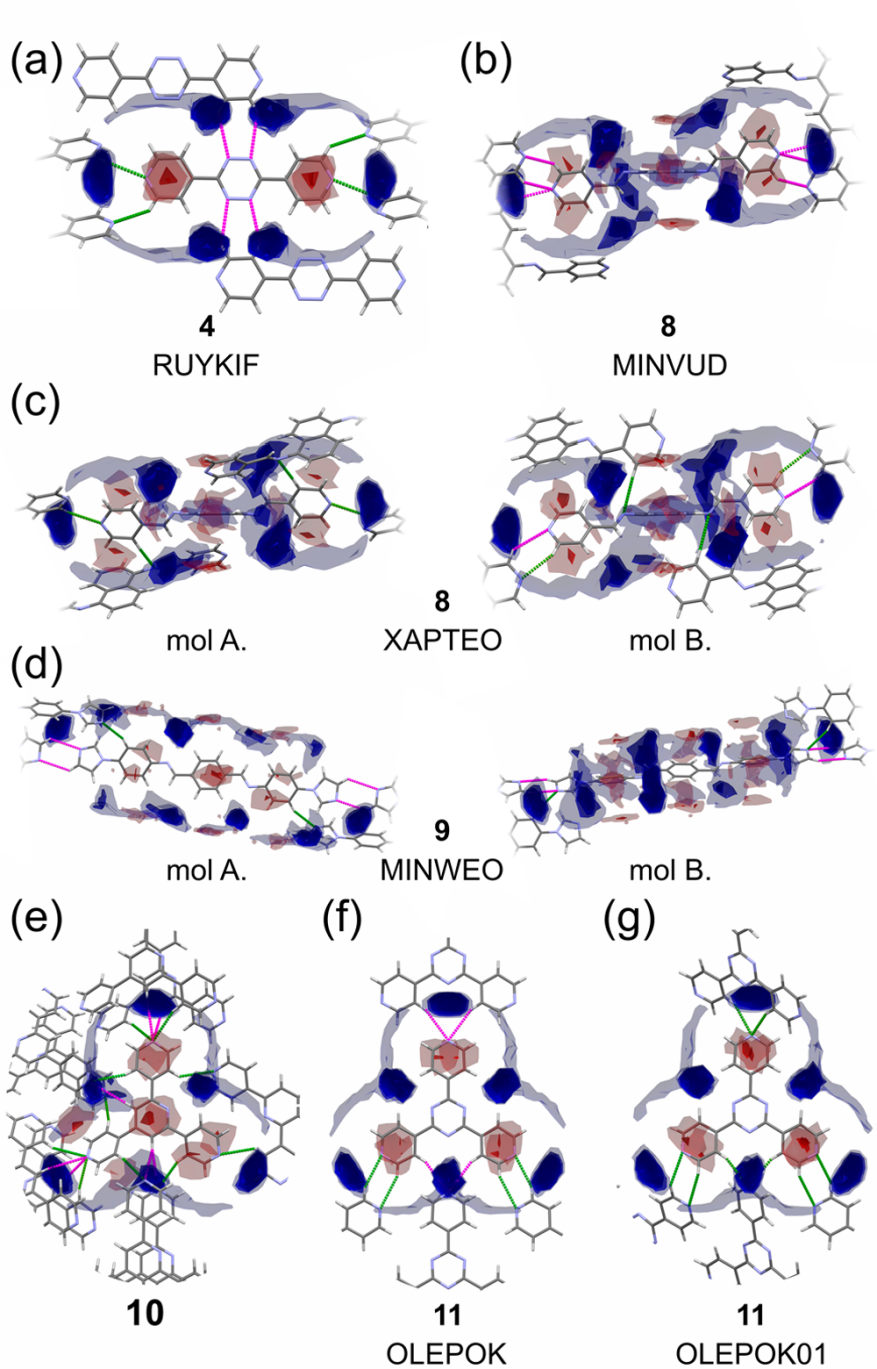
FIMs for anhydrate molecules: (a) **4** (RUYKIF), (b) **8** (MINVUD), (c) **8** (XAPTEO), (d) **9** (MINWEO), (e) **10**, (f) **11** (OLEPOK), and (g) **11** (OLEPOK01). For the color coding, see the caption for Figure 5. Possible very long contacts are marked in magenta. For the expanded FIM figures showing individual compounds, see Figures S21 and S23–S26 in the SI.

**Figure 8:**
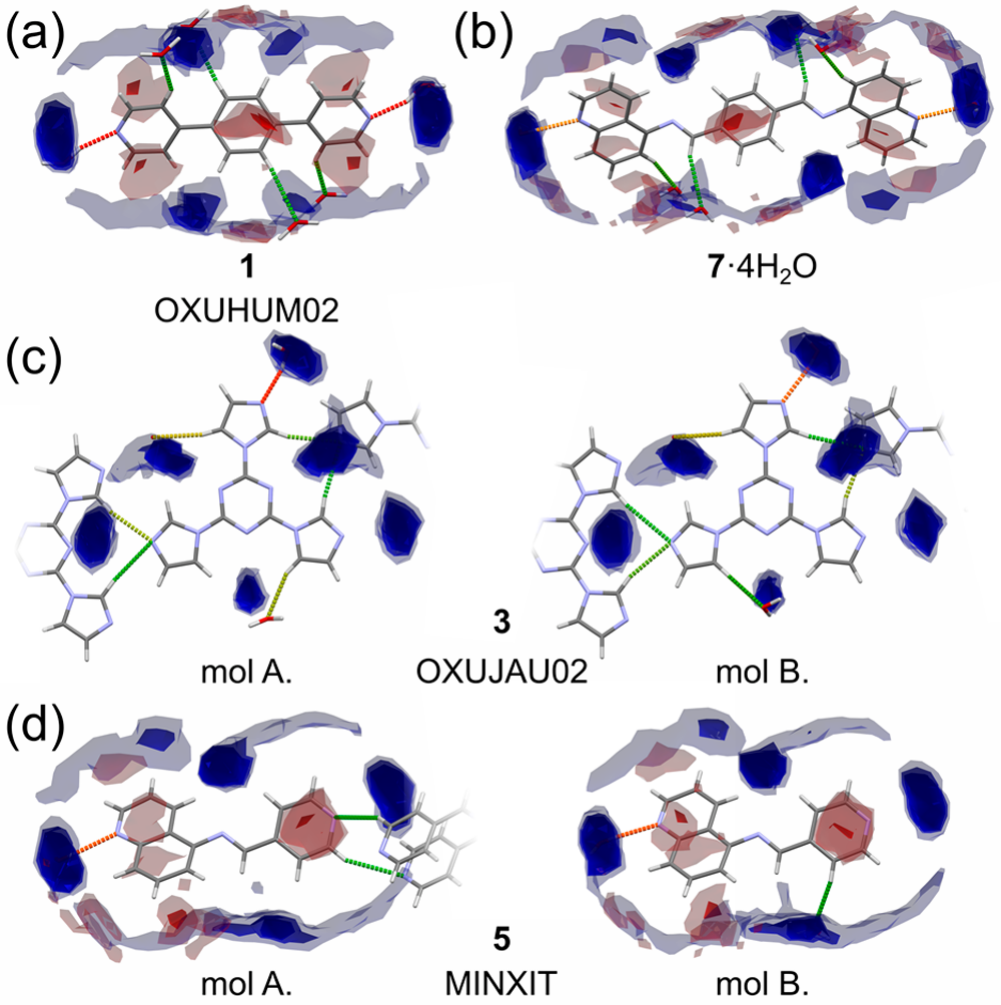
Interaction preferences for the molecular conformation of hydrate molecules: (a) **1** (OXUHUM02), (b) **7**, (c) **3** (OXUJAU02), and (d) **5**. For the color coding, see the caption for Figure 5. For an expanded version of the figure, see Figure S27 in the SI.

As discussed above in sections 3.2 and 3.3, electrostatic potential, molecular packing, and intermolecular contacts are all expected to affect the propensity for hydrate formation. In order to better understand the role of intermolecular interactions in hydrate formation, the hydrogen-bonding patterns present in known crystal forms of a given compound can be assessed against the intermolecular landscape statistically predicted for conformers present in anhydrate and hydrate crystals. Such a comparison is enabled by full interaction map (FIM) calculations. FIMs present regions where hydrogen-donor and hydrogen-acceptor functional groups are expected to be found, based on the geometry of intermolecular contacts present in crystal structures of similar organic compounds deposited in the CSD. Therefore, they can be used to evaluate how well the interaction preferences are fulfilled within a crystal structure. An advantage of FIMs over other computational methods used to explore interactions in the solid state is that an FIM requires little time. FIMs calculated for **1**–**11** (Figures 5–8) were compared in order to look for correlations between hydrate propensity and fulfilment of the landscape of expected intermolecular contacts.

Only **2** and **6** afforded crystal structures of both anhydrate and hydrate forms available for comparison. Interaction maps for the previously reported crystal structures of **2** are displayed in Figures 5a–c. **2** exists in three forms: anhydrate, monohydrate, and tetrahydrate (CSD refcodes PEXXEW, HIRLUQ, and OXUHIA02; respectively). In PEXXEW the asymmetric unit contains two crystallographically independent halves of **2**, shown as mol A and mol B. It is clear from Figure 5a that the existing hydrogen bonds between molecules of **2** either do not overlap with the regions where the presence of water oxygen atoms would be expected (mol B) or are at the edge of these regions (mol A). Meanwhile, FIMs of both hydrate forms of **2** revealed that water molecules are in the expected positions around molecules of **2** (Figures 5b and 5c). This comparison suggests that the geometry of intermolecular contacts in the anhydrate form of **2** does not satisfy the expected interaction preferences. However, incorporation of water molecules in the hydrate forms of **2** positions hydrogen donors near almost all electronegative regions of **2**. In the anhydrate form of **2**, mol A forms only two long contacts (shown in green) and mol B forms six long contacts for an average of four short contacts per molecule. Meanwhile, in the monohydrate (HIRLUQ) and tetrahydrate (OXUHIA02) forms of **2**, each molecule forms five and eight hydrogen bonds, respectively, most shorter than those in the anhydrate. The increased number of strong interactions could be the driving force for hydrate formation by **2**.

FIMs for both the hydrate and anhydrate forms of **6** are displayed in Figure 6. The anhydrate FIM revealed that hydrogen bonds between molecules of **6** lie outside or on the edge of the regions that are expected to be taken by the water molecules (Figure 6a). Similar to compound **2**, the water molecules in the dihydrate form of **6** increases the number of hydrogen donors and acceptors, as evidenced by FIMs (Figure 6b).

The FIMs for the molecules that did not form hydrates during screening experiments (**4**, **8**, **9**, **10**, and **11**) are presented in Figure 7. Three structural features were observed, based on the FIMs analysis of compounds **4**, **8**, **10**, and **11**. First, the intermolecular contacts formed between N-heterocyclic molecules are geometrically coherent with or are very close to the regions expected to be occupied by water molecules (RUYKIF, XAPTEO mol A, OLEPOK01). Second, the number of hydrogen bonds, longer intermolecular contacts, and C–H**···**π interactions per molecule is relatively high (**4**, XAPTEO, MINWEO, **10**, OLEPOK, OLEPOK01). Finally, the hydrogen-bonding pattern is restricted by the shape of the molecule (RUYKIF, **10**, OLEPOK, OLEPOK01). Interestingly, in the cases of **4**, **10**, and **11** molecular shape allows for tight packing and formation of long contacts around the regions expected to be occupied by water molecules. Although, these contacts are long, their number is higher than the number of hydrogen bonds formed if the same regions were occupied by individual water molecules. The propensity of **9** to exist as anhydrate can be attributed to the large number of weak interactions in the crystal packing of **9** (see Table S2 in the SI). For clarity, only selected contacts are shown in FIMs for MINWEO, illustrating how each symmetrically independent molecule is involved in only two hydrogen bonds, and four additional long intermolecular contacts. Nevertheless, note that molecules of **9** are also involved in numerous C–H**···**π interactions (Table S2) not shown in the FIM. Based on these observations, it can be suggested that hydrate formation for **4**, **8**, **9**, **10**, and **11** was prevented because molecules were able to pack with hydrogen-bonding patterns competitive to those feasible in the presence of water molecules. The only outlier in this group is MINVUD, in which the intermolecular landscape is unfulfilled. However, it is the least-stable polymorph of **8**. The other polymorph, XAPTEO, exhibits a more favorable hydrogen-bonding pattern.

In the case of those molecules for which only the crystal structures of hydrates are known (**1**, **3**, **5**, and **7**), the FIMs are presented in Figure 8. As expected, in all cases, water molecules are positioned as anticipated from the FIMs, resulting in geometrically favored hydrogen-bonding patterns.

Based on the analysis of the FIMs, we can suggest that incorporation of water molecules occurs to obtain geometrically favored interactions that are not possible in the corresponding anhydrate forms. Conversely, if molecules can achieve a comparable outcome by efficient packing, in some cases enabled by adjusting conformation, then anhydrate forms can be favored.

## 4 Conclusions

We report herein a study of hydrate propensity in 5- and 6-membered *N*-heterocyclic aromatics that lack strong hydrogen-bond donors. Our investigation involved a CSD survey, systematic hydrate screening experiments, analyses of electrostatic potential maps, full interaction maps, and crystal packing. We observe that the relationship between hydrates and anhydrates can be influenced by multiple factors, including the experimental technique used for hydrate formation. In particular, for **2**, DVS was not as suitable as the other techniques that were used.

Regarding the questions raised in the Introduction, the following conclusions about the group of molecular compounds studied herein can be drawn:

(i) hydrate propensity did not increase with the increase in the number of hydrogen-bond acceptors;
(ii) no correlation was found between the number of hydrogen-bond acceptors and the stoichiometry of water in the crystal lattice; and
(iii) crystal packing can play a significant role in determining hydrate propensity and is influenced by two factors: the number of weak intermolecular interactions in an anhydrate and whether or not a molecular conformation results in an optimal electrostatic potential distribution.

With respect to item (iii), this study suggests that analysis of FIMs can be a powerful tool not only for the study of polymorphs but also to provide insight into hydrate propensity. Analysis of FIMs suggested that hydrate formation is inhibited when molecules of a given compound can achieve geometrically favorable hydrogen-bonding patterns on their own. In cases where formation of molecular aggregation is unfavorably affected by shape and conformation, incorporation of water molecules can lead to efficient crystal packing by involving water molecules. However, we emphasize that the number of crystal structures analyzed in this study is limited and a more extensive study would be required to assess the generality of this finding.

The results obtained from this study once more lead us to the conclusion that hydrate formation is very hard to predict and that hydrates can indeed be described as a nemesis to crystal engineering.^6^ Nevertheless, systematic studies similar to that presented herein may bring us closer to a better understanding of at least some aspects of their formation.

## Supporting Information Available

PXRD and thermal analyses, detailed crystallographic information and additional figures (PDF)

## Accession Codes

CCDC 1897070, 1859837, and 1859838 contain the supplementary crystallographic data for this paper. These data can be obtained free of charge via www.ccdc.cam.ac.uk/data_request/cif, or by emailing data_request@ccdc.cam.ac.uk, or by contacting The Cambridge Crystallographic Data Centre, 12 Union Road, Cambridge CB2 1EZ, UK; fax: +44 1223 336033.
