# Supplementary material for: Toward an Understanding of the Propensity for Crystalline Hydrate Formation by Molecular Compounds. Part 2
Source: Cryst Growth Des. 2021 Jul 30;21(9):4927–39. doi: 10.1021/acs.cgd.1c00353 (PMC8414477; doi:10.1021/acs.cgd.1c00353)
Supplement: Supplementary file 1 — cg1c00353_si_001.pdf [file cg1c00353_si_001.pdf]

Supplementary Information

Towards an understanding of the propensity for crystalline hydrate formation by molecular compounds. Part 2

Rana Sanii,[a] Ewa Patyk-Kaźmierczak,[a,b] Carol Hua,[c] Shaza Darwish,[a] Tony Pham,[d] Katherine A. Forrest,[d] Brian Space,[d] and Michael J. Zaworotko[a*]

[a] Department of Chemical Sciences and Bernal Institute, University of Limerick, Co. Limerick, Y94T9PX, Ireland.
[b] Department of Materials Chemistry, Faculty of Chemistry, Adam Mickiewicz University, Uniwerystetu Poznańskiego 8, 61-614, Poznań, Poland.
[c] School of Chemistry, University of Melbourne, Victoria, 3010, Australia.
[d] Department of Chemistry, University of South Florida, 4202 East Fowler Avenue, Tampa, Florida, 33620, U.S.A.

Table of Contents

**1.**	The library of N-heterocyclic compounds	2
**2.**	Hydrates in the context of pharmaceutical industry	3
**3.**	Synthesis of Compounds 1-11	**4**-5
**4.**	Slurry Experiments of Compounds 4-11	5-6
**5.**	Characterization of compounds 1-11	7-15
**6.**	Crystal Data and Refinement Details	16,17
**7.**	Intermolecular interactions present in the crystal structures of compounds 1-11	18-21
**8.**	Full Interaction Maps of Compound 1-11	22-28
**9.**	List of dihedral and torsion angles of compounds 1-11	**29**-32
**10.**	References	33-34

Scheme 1. The library of N-heterocyclic compounds investigated herein for their propensity to form hydrates. REFCODEs for structures reported in the CSD of anhydrate (Anh) and hydrate (H2O) forms, and previously unreported structures (New) are listed.

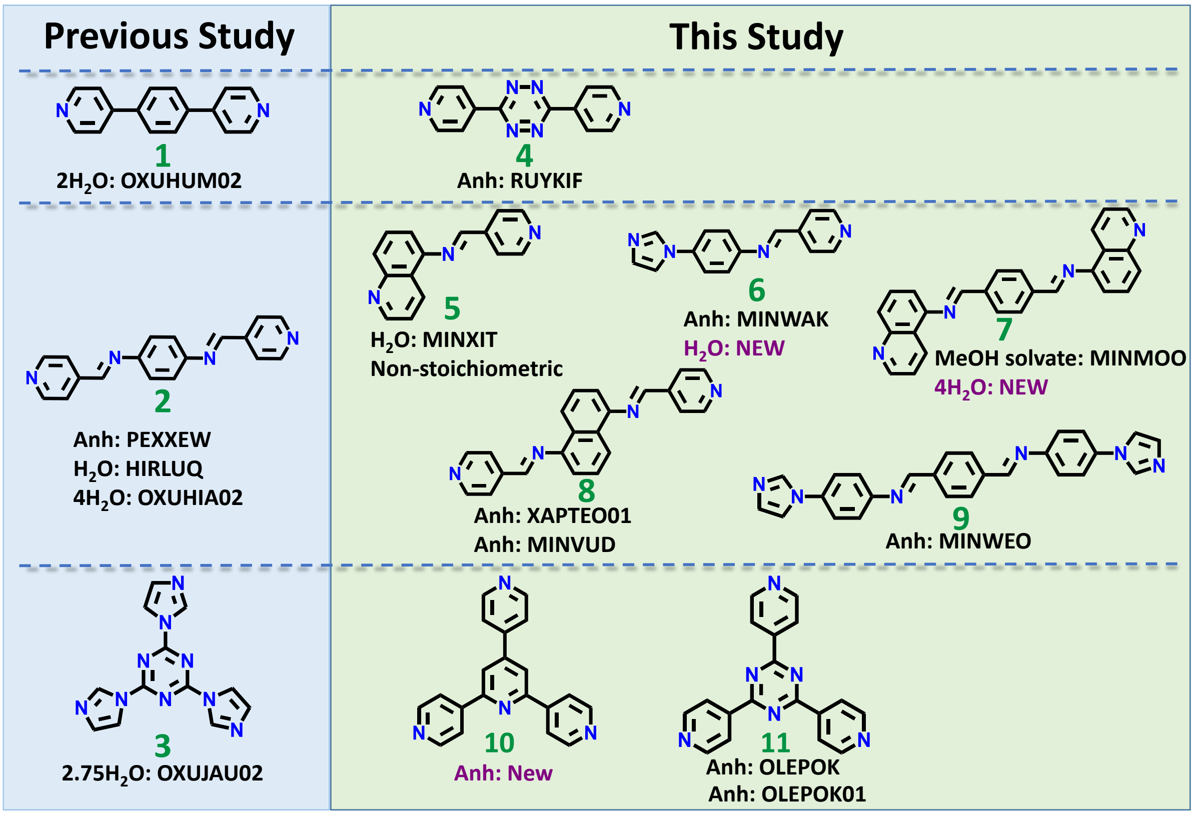

Hydrates in the context of pharmaceutical industry

In orally delivered drug products, active drug substances are almost always used as crystalline solids because of the inherent stability and purity of crystal forms, reproducibility of properties and the scaleability of crystallization processes. There is strong motivation for pharmaceutical scientists to study the range of crystal forms that exist for drug compounds, as the physicochemical properties of a drug substance can be affected by its solid form.[1-5] Routine crystallization screening can be used to identify those crystal form(s), typically polymorphs, solvates or hydrates, with properties suitable for use in a drug product. These screening experiments, which can be conducted robotically, also facilitate setting of the parameters needed to control the formation of a specific crystal form during manufacturing.[6] The latter issue is especially important in the context of hydrates, as the small size of water molecules and its multi-faceted H-bonding capability can favor hydrate formation. Further, the ubiquity of water vapor means that hydrates can form spontaneously.[2,7-10]

Formation of a hydrate is not necessarily a problem as a hydrate can ultimately be more suited for use in a drug product than an anhydrate.[11] Indeed, the presence of water in a drug substance does not raise any serious regulatory concerns as there are no toxicology risks associated with its presence in the resulting drug product. Hydrate formation can, however, impact intermolecular interactions and alter the physicochemical properties (e.g. solubility,[12] hygroscopicity,[13] stability[14] and bioavailability[11]) of a drug substance.[2,15] In addition, a change in the hydration state of a drug substance may impact mechanical behavior during tableting or grinding and thereby affect product performance.[4,15,16] Nevertheless, a hydrated solid form does not limit the use of a drug substance in a marketed drug product.[17] Indeed, identification of hydrated crystal forms is a step in drug development.[15,18] Overall, hydrates are often used in solid drug products, either as the active ingredient or an inactive excipient.[15,19,20] A survey of the literature has revealed that drug products based upon hydrates include creatine phosphate sodium,[21] morphine sulphate,[22] azithromycin,[23] erythromycin,[24] amoxicillin,[25] fosamax,[25] lipitor,[25] protonix,[25] darunavir,[26] lisinopril,[27] cefaclor,[28] ampicillin,[29] cephalexin,[30] cefadroxil,[31] theophylline,[32,33] nitrofurantoin[34] and paroxetine hydrochloride.[35] However, there are also cases where formation of a solvate or hydrate renders the substance less suitable for use in a drug product as exemplified by paracetamol, cimetidine or naproxen, the active ingredients of the respective over-the-counter (OTC) drug products Tylenol (Panadol), Tagamet and Aleve. Mometasone furoate, pazopanib and seratraline hydrochloride, present in prescription medicines, Elocon, Votrient, and Zoloft, respectively, also have concerns related to hydrate formation.[25,36]

The process used to manufacture a formulated drug product can also be impacted by polymorpism or pseudopolymorphism in its excipients.[15] Hydrate formation by excipients such as lactose, glucose, magnesium stearate or calcium phosphate are among examples studied in the literature.[15,19] Control over hydrate and solvate formation in molecular compounds is therefore of particular interest to pharmaceutical science, where one-third of drug substances are thought to form crystalline hydrates.[18,36] Indeed, 31.9% of entries in the European Pharmacopeia (1991) are hydrates and 11.2% are solvates.[37] Similar statistics were reported in 1999 for organic compounds in general, with hydrates more prelevant (33%) than solvates (10%).[38] A study by Rodriguez-Spong *et al*. based on a survey of the Cambridge Structural Database (CSD) also indicated that hydrate formation for organic compounds occurs more frequently than solvate formation with organic solvents.[39]

Synthesis of Compounds 1-11

1,4-Bis(4-pyridyl)benzene (1). Compound **1** was synthesised following a previously published method by our group by 2-fold Pd0-catalyzed Suzuki coupling of 4-pyridinylboronic acid with 1,4-dibromobenzene.[40] A 250 mL oven-dried two-necked round bottom flask was cooled under N2 atmosphere and charged with 1,4-dibromobenzene (1.61 g, 6.85 mmol), 4-pyridinylboronic acid (2.52 g, 20.5 mmol), $Pd(PPh_3)_4$ (0.39 g, 0.42 mmol), powdered NaOH (1.10 g, 27.4 mmol), 30 mL of toluene, 20 mL of EtOH and 10 mL of distilled water. The resultant reaction mixture was refluxed at 110 ^oC. The contents dissolved completely to give clear yellow coloration over a period of 1.5 h. Heating was continued at reflux under N_2 atmosphere for 2 d. The change in the color of reaction mixture from yellow to dark brown indicated completion of the reaction, which was further verified by TLC. Subsequently, the reaction mixture was cooled and extracted with $CHCl_3$ and washed with brine solution. The organic phase was dried over anhyd Na_2SO_4 and concentrated *in vacuo*. The pure product was isolated by Silica gel column chromatography using $CHCl_3$/pet. ether (40%) mixture as an eluent to afford **1** as a white solid in 96% yield.

Bis(Pyridin-4-ylmethylene)benzene-1,4-diamine (2). Compound **2** was prepared following a previously published method by our group.[41] p-phenylenediamine (0.11 g, 1.0 mmol) and 4-pyridinecarboxaldehyde (0.21 g, 2.0 mmol) were ground until a free-flowing yellow powder was obtained. Upon addition of ~100 µL of MeOH the resultant yellow paste was further ground (ca. 10 min) until a free-flowing deep yellow powder was obtained as pure product in 95.4% yield.

2,4,6-Tris(imidazol-1-yl)-1,3,5-s-triazine (3). Compound **3** was obtained following a previously reported nucleophilic substitution of cyanuric chloride with imidazole under solvent free conditions reported by Azarifar *et al*.[42]

3,6-Di(pyridin-4-yl)-1,2,4,5-tetrazine (4). Compound **4** was prepared following a previously published method.[43] Compound **4** was obtained by mixing 4-cyanopyridine (0.10 g, 1.0 mmol), hydrazine hydrate (0.16 g, 5.0 mmol), nickel catalyst (0.5 mol %) and sulphur (64 mg, 2.0 mmol) in 3 mL ethanol at 78 °C and stirring the mixture for 24 h in 83% yield.

***N*-(Pyridin-4-ylmethylene)quinolin-5-amine (5).** Compound **5** was prepared following a previously published method by our group.[41] 5-Aminoquinoline (0.14 g, 1.0 mmol) and 4-pyridinecarbxaldehyde (0.10 g, 1.0 mmol) were ground until a free-flowing yellow powder was obtained. Upon addition of ~100 µL of MeOH the resultant yellow paste was further ground (ca. 10 min) until a free-flowing deep yellow powder was obtained as pure product in 98% yield.

***N,N*'-4-(1H-imidazol-1-yl)-*N*-(pyridin-4-ylmethylene)aniline (6).** Compound **6** was prepared following a previously published method by our group.[41] 4-(1H-imidazol-1-yl)aniline (0.16 g, 1.0 mmol) and 4-Pyridinecarboxaldehyde (0.11 g, 1.0 mmol) were ground until a free-flowing powder was obtained. Upon addition of ~100 µL of 2-propanol the resultant paste was further ground (*ca.* 10 min) until a free-flowing light brown powder was obtained as pure product in 97% yield.

***N,N'*-(1,4-phenylenebis(methan-1-yl-1-ylidene))diquinolin-5-amine (7).** Compound **7** was prepared following a previously published method by our group.[41] Terephthalaldehyde (0.13 g, 1.0 mmol) and 5-aminoquinoline (0.23 g, 2.0 mmol) were ground into the fine powder. Subsequently, ~100 µL of

MeOH was added and further ground until the paste turned into a free-flowing yellow powder (*ca.*10 min). The yellow solid was heated at 150 ˚C for 2 h to isolate the pure anhydrous product in 97% yield.

***N,N*'-Bis(4-pyridylmethylene)naphthalene-1,5-diamine (8).** Compound **8** was prepared following a previously published method by our group.[41] 1,5-Diaminonaphthalene (0.08 g, 0.5 mmol) and 4-pyridinecarboxaldehyde (0.11 g, 1.0 mmol) were ground until a free-flowing yellow powder was obtained. Upon addition of ~100 µL of MeOH the resultant yellow paste was further ground (*ca.* 10 min) until a free-flowing deep yellow powder was obtained as pure product in 97% yield.

***N,N'*-(1,4-phenylenebis(methan-1-yl-1-ylidene))bis(4-(1H-imidazol-1-yl)aniline) (9).** Compound **9** was prepared following a previously published method by our group.[41] Terephthalaldehyde (0.13 g, 1.0 mmol) and 4-(1H-imidazol-1-yl)aniline (0.31 g, 2.0 mmol) were ground until a free-flowing powder was obtained. Upon addition of ~100 µL of MeOH the resultant paste was further ground (*ca.* 10 min) until a free-flowing deep yellow powder was obtained in 98% yield.

2,4,6-Tris(4-pyridyl)pyridine (10). Compound **10** was prepared by the cyclization reaction of 4-acetylpyridine and 4-pyridinecarbaldehyde following a previously published method by our group (56% yield).[44]

2,4,6-Tris(4-pyridyl)-1,3,5-triazine (11). Compound **11** was synthesised following a previously published method from the catalytic transformation of 4-cyanopyridine in presence of the magnesium phthalocyanine as catalyst.[45]

Slurry Experiments of Compounds 4-11

General aspect

Slurry experiments of **4**-**11** were performed under ambient conditions. 50 mg of each compound was slurried in pure water up to 7 days. The volume of solvent used to suspend the sample was one-third of the volume required to dissolve it completely. Powder X-ray diffraction (PXRD) and thermogravimetric analyses (TGA) were used to determine the hydrate formation. To examine the relative stability of the isolated hydrates, mixtures of hydrate and anhydrate forms in 1:1 w/w ratio were slurried in mixed solvent system of EtOH and H_2O in varied EtOH:H_2O vol. ratios: 1:1, 5:1 and 1:5.

3,6-Di(pyridin-4-yl)-1,2,4,5-tetrazine (4). 50 mg of **4** was slurried in water at room temperature in a sealed glass vial. Aliquots of sample were removed after 7 days in order to record the PXRD and TGA patterns (Figures S2 and S3).

***N*-(Pyridin-4-ylmethylene)quinolin-5-amine (5).** 50 mg of **5** was slurried for 24 h in water at room temperature in a sealed glass vial. Competitive slurry was conducted using 50 mg of the mixture of hydrate and anhydrate of **5** in a 1:1 w/w ratio, and 1:1, 5:1 and 1:5 EtOH:H_2O solvent system. Aliquots of sample were removed after 24 h in order to record the PXRD and TGA patterns (Figures S4 and S5).

***N,N*'-4-(1H-imidazol-1-yl)-*N*-(pyridin-4-ylmethylene)aniline (6).** 50 mg of **6** was slurried for 24 h in water at room temperature in a sealed glass vial. Competitive slurry was conducted using 50 mg of the mixture of hydrate and anhydrate of **6** in a 1:1 w/w ratio, and 1:1, 5:1 and 1:5 EtOH:H_2O solvent system. Aliquots of sample were removed after 24 h in order to record the PXRD and TGA patterns (Figure S6 and S7).

***N,N'*-(1,4-phenylenebis(methan-1-yl-1-ylidene))diquinolin-5-amine (7).** 50 mg of **7** was slurried for 24 h in water at room temperature in a sealed glass vial. Competitive slurry was conducted using 50 mg of the mixture of hydrate and anhydrate of **7** in a 1:1 w/w ratio, and 1:1, 5:1 and 1:5 EtOH:H2O solvent system. Aliquots of sample were removed after 24 h in order to record the PXRD and TGA patterns (Figures S8 and S9).

***N,N*'-Bis(4-pyridylmethylene)naphthalene-1,5-diamine (8).** 50 mg of **8** was slurried in water at room temperature in a sealed glass vial. Aliquots of sample were removed after 7 days in order to record the PXRD and TGA patterns (Figures S10 and S11).

***N,N'*-(1,4-phenylenebis(methan-1-yl-1-ylidene))bis(4-(1H-imidazol-1-yl)aniline) (9).** 50 mg of **9** was slurried in water at room temperature in a sealed glass vial. Aliquots of sample were removed after 7 days in order to record the PXRD and TGA patterns (Figures S12 and S13).

2,4,6-Tris(4-pyridyl)pyridine (10). 50 mg of **10** was slurried in water at room temperature in a sealed glass vial. Aliquots of sample were removed after 7 days in order to record the PXRD and TGA patterns (Figures S14 and S15).

2,4,6-Tris(4-pyridyl)-1,3,5-triazine (11). 50 mg of **11** was slurried in water at room temperature in a sealed glass vial. Aliquots of sample were removed after 7 days in order to record the PXRD and TGA patterns (Figures S16 and S17).

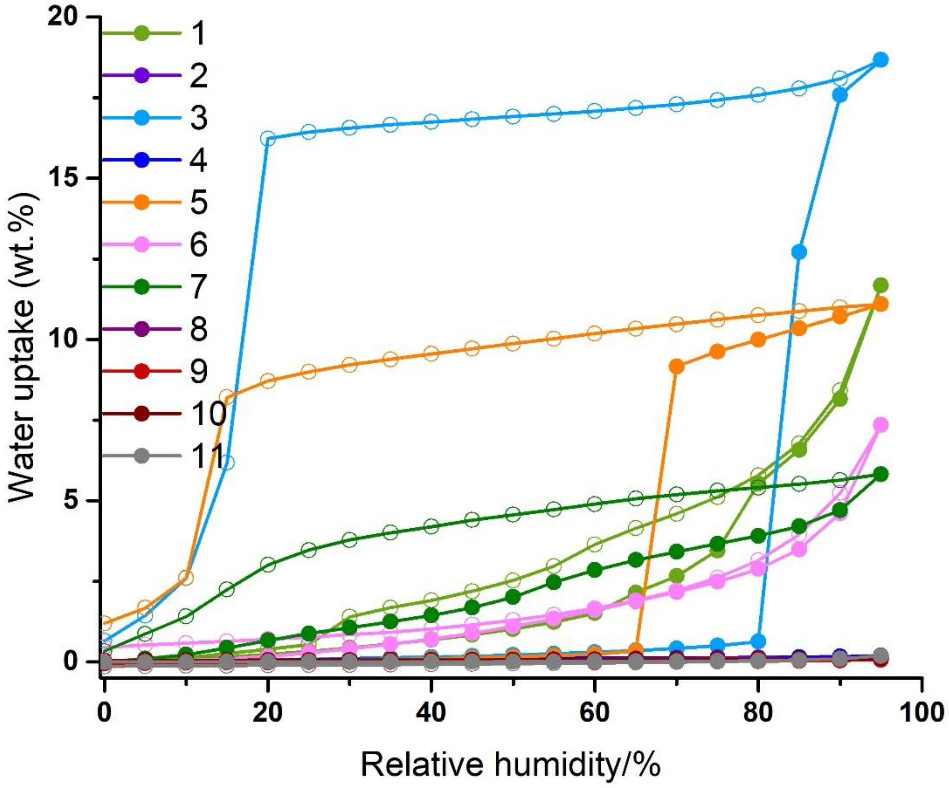

Figure S1. DVS data for anhydrates of compounds **1-11**. Adsorption and desorption are shown in solid and hollow circles, respectively.

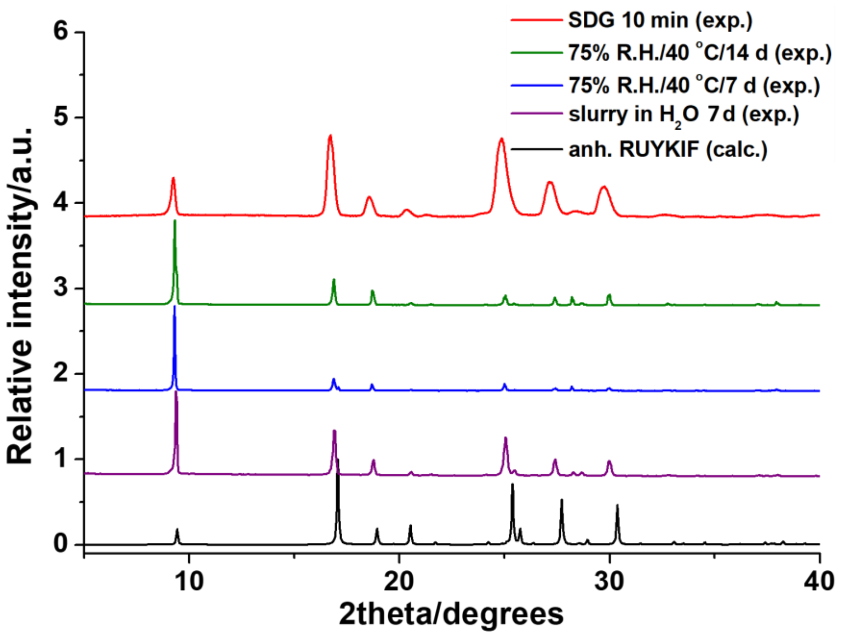

Figure S2. PXRD patterns for **4** after hydrate screening experiments.

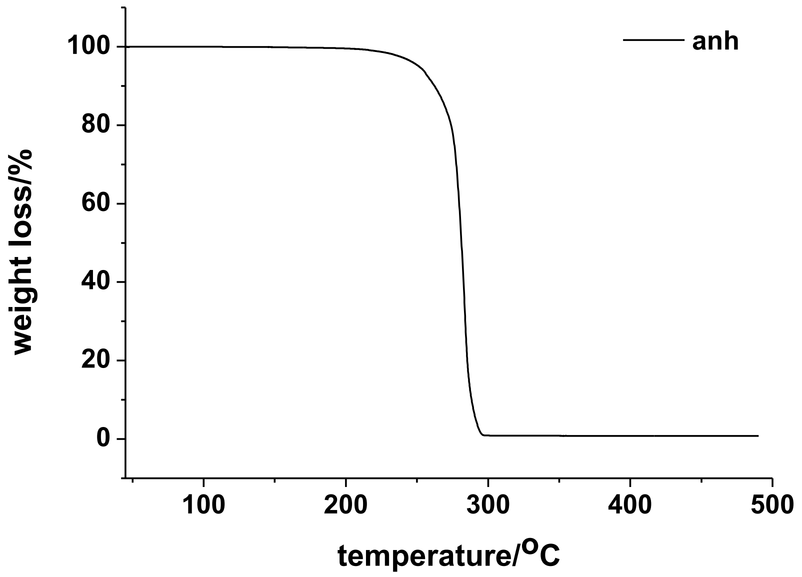

Figure S3. TGA profile of **4** after slurry in water at room temperature (rt) for 7 d.

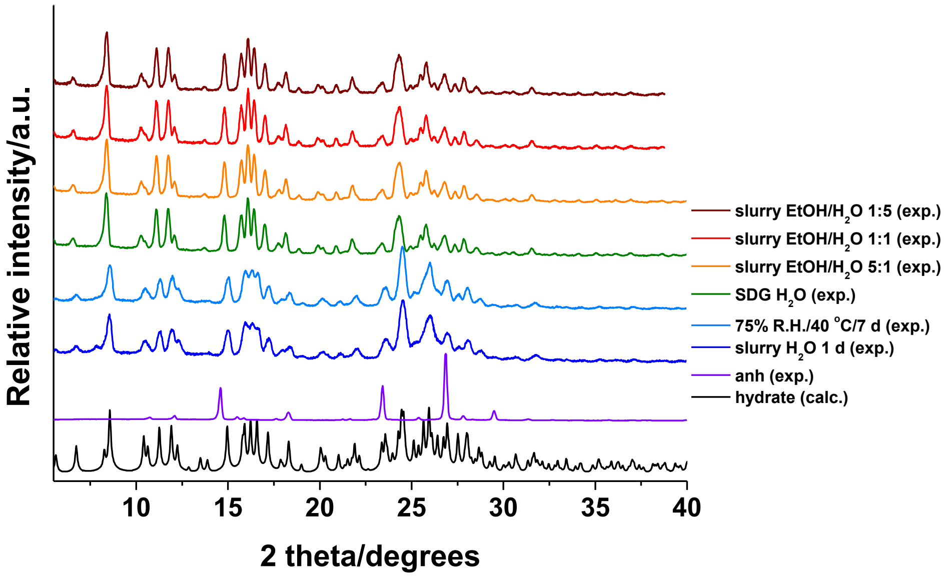

Figure S4. PXRD patterns for **5** after hydrate screening experiments.

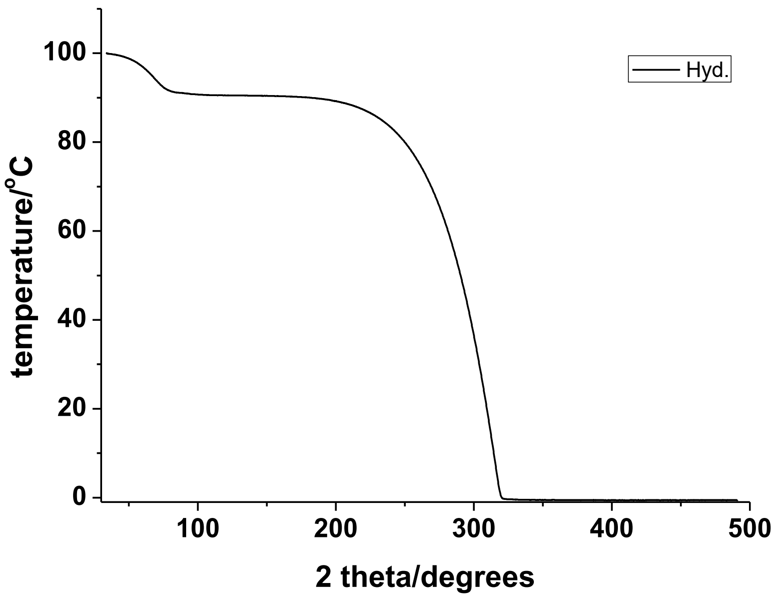

Figure S5. TGA profile of **5** after slurry in water at rt for 1 d.

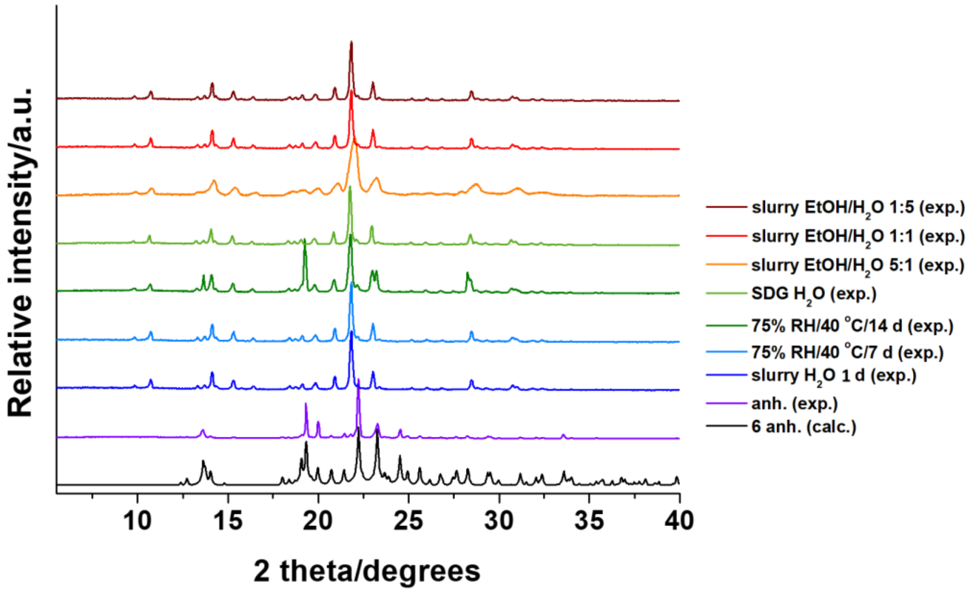

Figure S6. PXRD patterns for **6** after hydrate screening experiments.

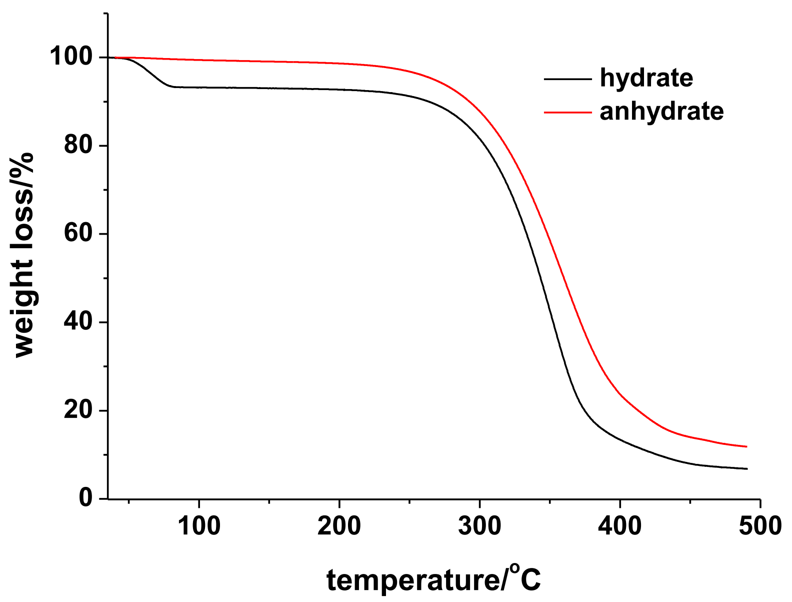

Figure S7. TGA profiles for the hydrate and anhydrous forms of **6**. TGA profile of hydrated form of **6** (marked in black) was collected for sample slurried in water at rt for 1d.

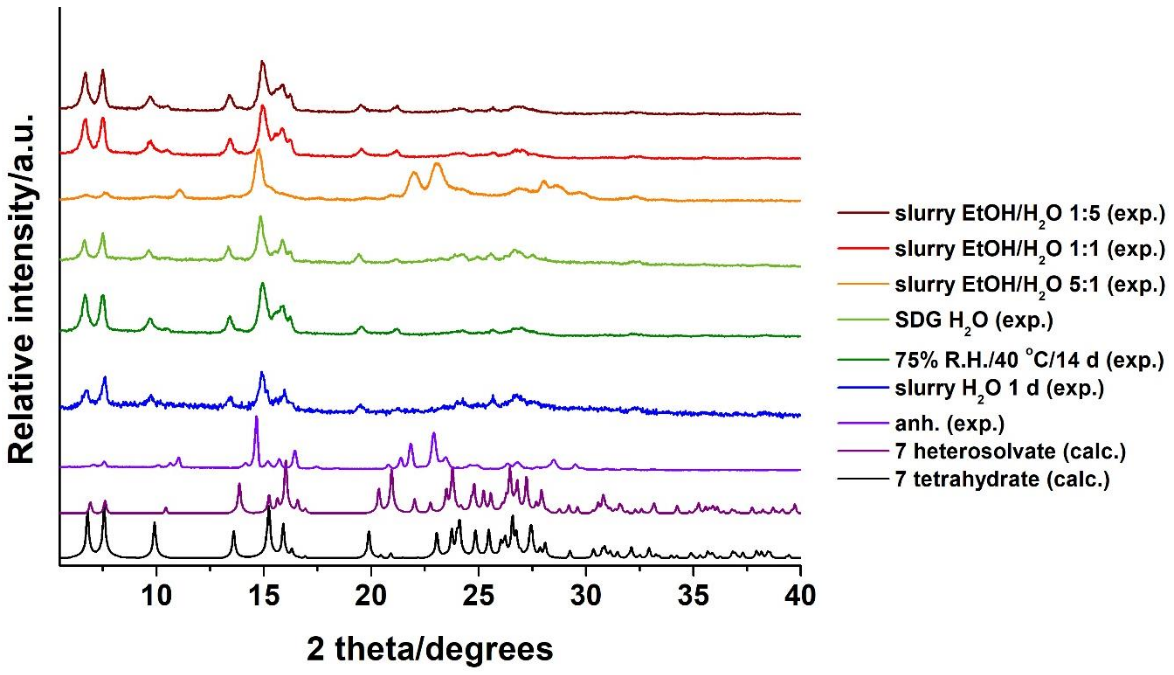

Figure S8. PXRD patterns for **7** after hydrate screening experiments.

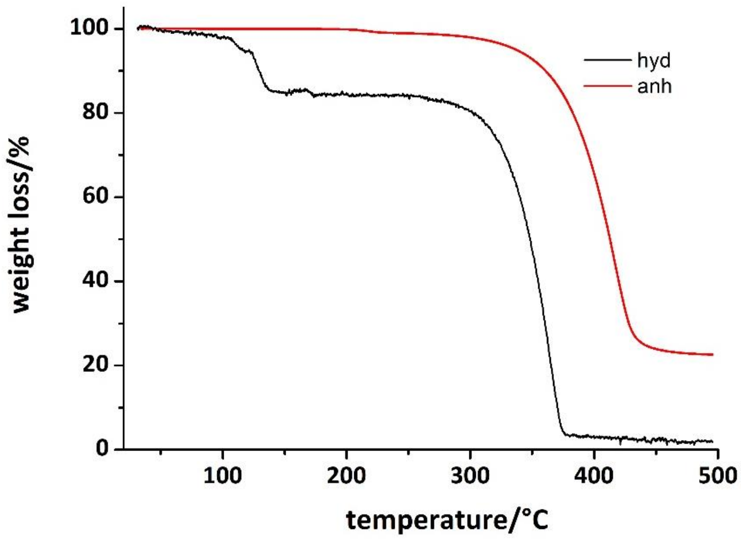

Figure S9. TGA profiles for the hydrate and anhydrous forms of **7**. TGA profile of hydrated form of **7** (marked in black) was collected for sample slurried in water at rt for 1 d.

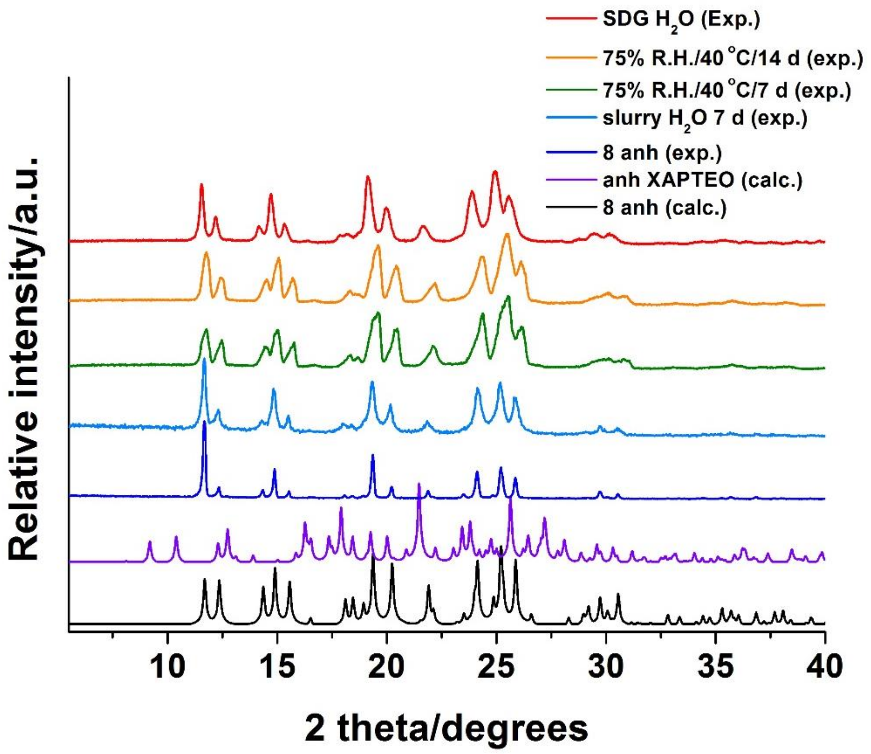

Figure S10. PXRD patterns for **8** after hydrate screening experiments.

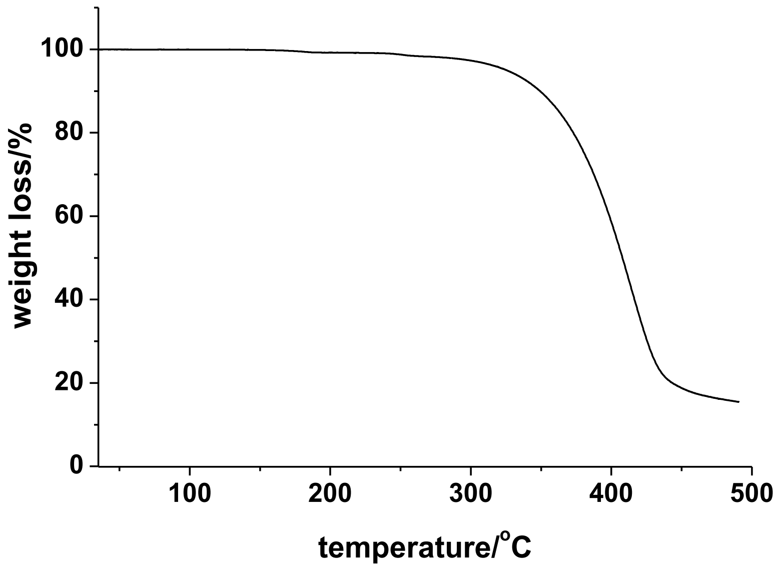

Figure S11. TGA profile of **8** after slurry in water at rt for 7 d.

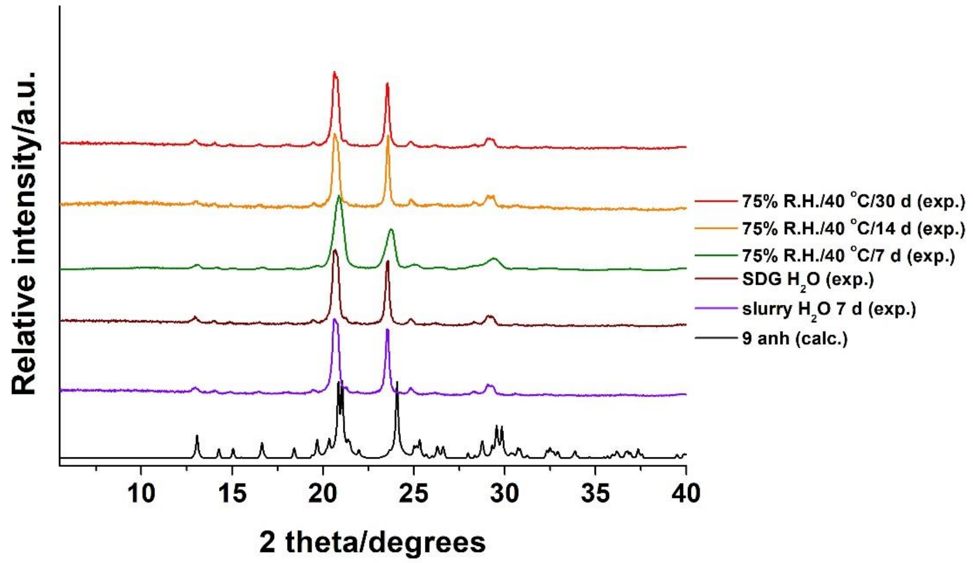

Figure S12. PXRD patterns for **9** after hydrate screening experiments.

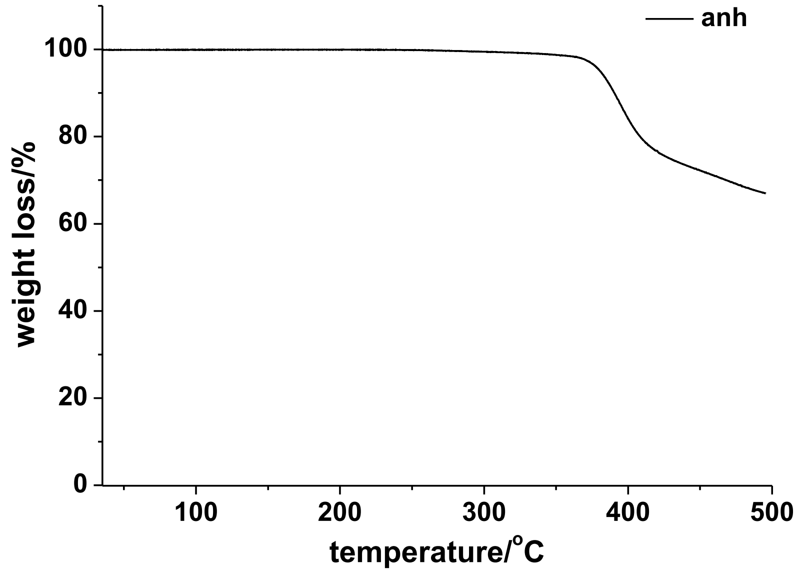

Figure S13. TGA profile of **9** after slurry in water at rt for 7 d.

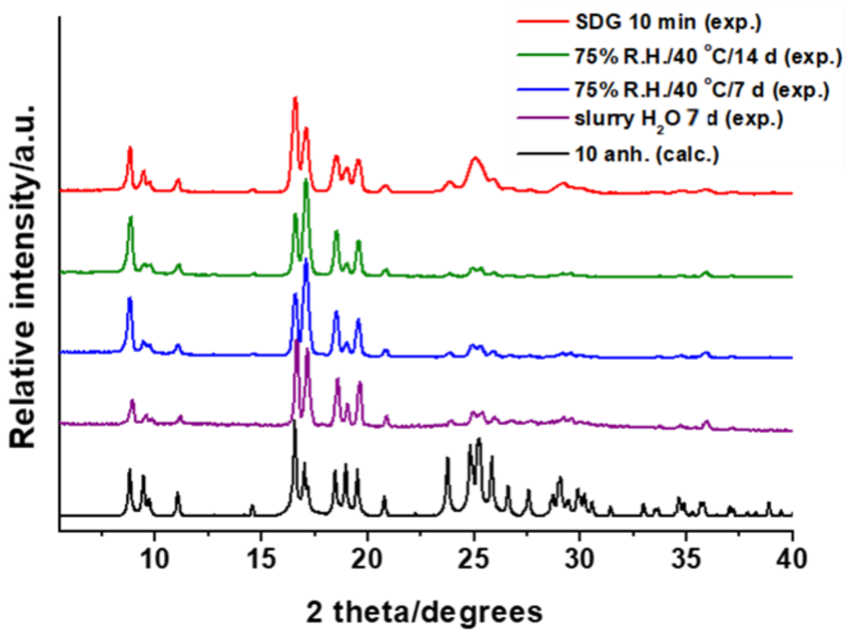

Figure S14. PXRD patterns for **10** after hydrate screening experiments.

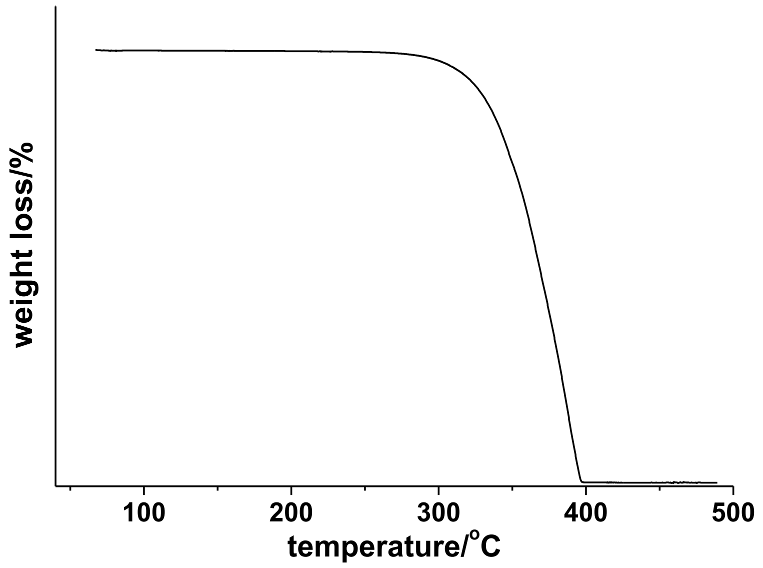

Figure S15. TGA profile of **10** after slurry in water at rt for 7 d.

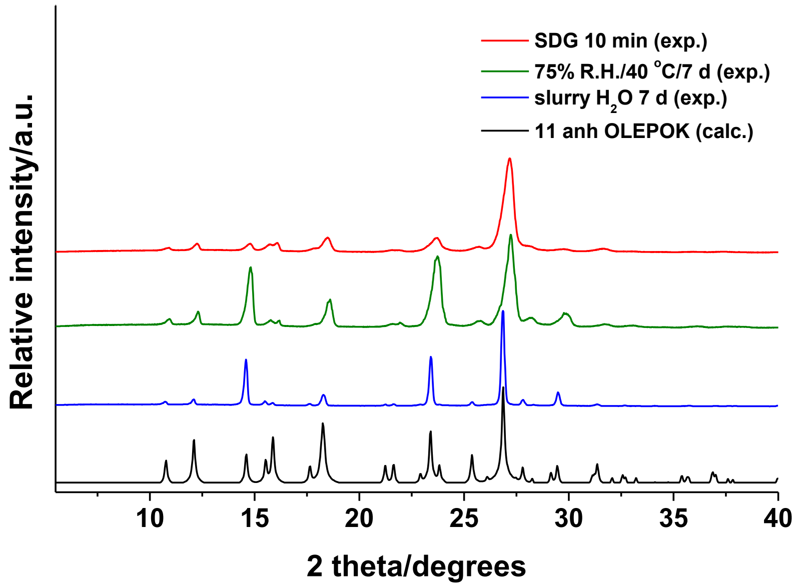

Figure S16. PXRD patterns for **11** after hydrate screening experiments.

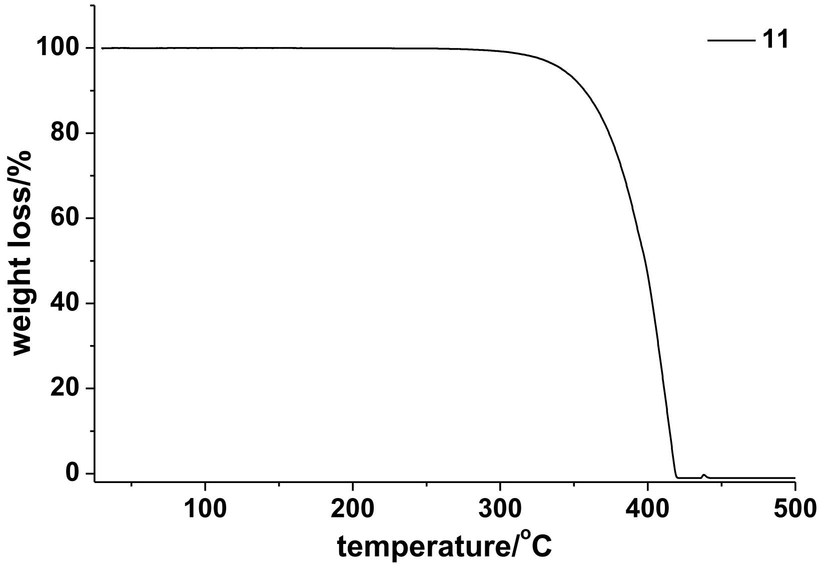

Figure S17. TGA for **11** after slurry in water for 7 d.

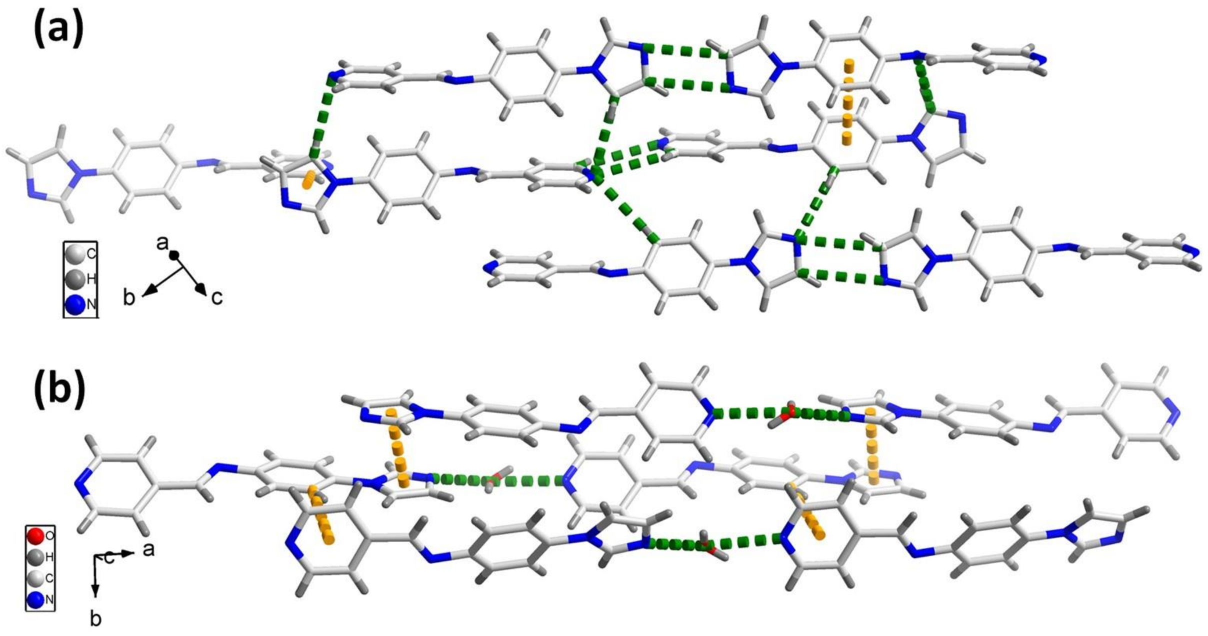

Fig S18. Crystal structures depicting multiple intermolecular interactions (C-H···N and O-H···N in green; C-H···π and π-π in yellow) in the anhydrate of **6** (a) and hydrate of **6** (b).

Table S1. Crystal Data and Refinement Details

a. For structures determined based on single-crystal diffraction data

Compound	**7**·4H_2O	**10**
Formula	$C_{26}H_{18}N_4$•4H_2O	$C_{20}H_{14}N_4$
MW (g·mol^{-1})	458.51	310.35
T (K)	273(2)	100(2)
Crystal system	Monoclinic	Monoclinic
Space group	$P2_1/c$	$P2_1/c$
Z	2	4
a (Å)	13.032(3)	3.7393(2)
b (Å)	3.9117(8)	10.3779(5)
c (Å)	23.435(5)	37.1842(17)
α (°)	90	90
β (°)	92.697(11)	90.191(3)
γ (°)	90	90
V (Å^3)	1193.4(4)	1442.97(12)
ρ_{calc} (g·cm^{-3})	1.276	1.429
μ (mm^{-1})	0.714	0.693
Measured/independent reflections (R_{int})	16128/1747 (0.0757)	10746/2099 (0.0550)
Observed reflections [I > 2σ(I)]	1453	1985
R_1, wR_2 [I > 2σ(I)]	0.0973, 0.2479	0.0612, 0.1544
R_1, wR_2 (all data)	0.1078, 0.2657	0.0641, 0.1565
$\Delta\rho_{min}$, $\Delta\rho_{max}$ (e Å^{-3})	-0.298, 0.338	-0.270, 0.234
Goodness-of-fit on F^2	1.086	1.091

b. For structure determined based on powder diffraction data

Compound	**6**·H_2O
Formula	$C_{15}H_{12}N_4$·H_2O
MW (g·mol^{-1})	266.305
T (K)	300(2)
Crystal system	Monoclinic
Space group	$P2_1/a$
Z	4
a (Å)	16.715(9)
b (Å)	9.2843(15)
c (Å)	9.107(4)
β (°)	99.406(18)
V (Å^3)	1394.3(8)
ρ_{calc} (g·cm^{-3})	1.268
R_p(%)	7.64
R_{exp}(%)	6.94
R_F(%)	7.28
R_{F2}(%)	4.70
wR_p(%)	9.82
R_B (%)	7.26
wR_B (%)	8.34
Goodness-of-fit	1.934

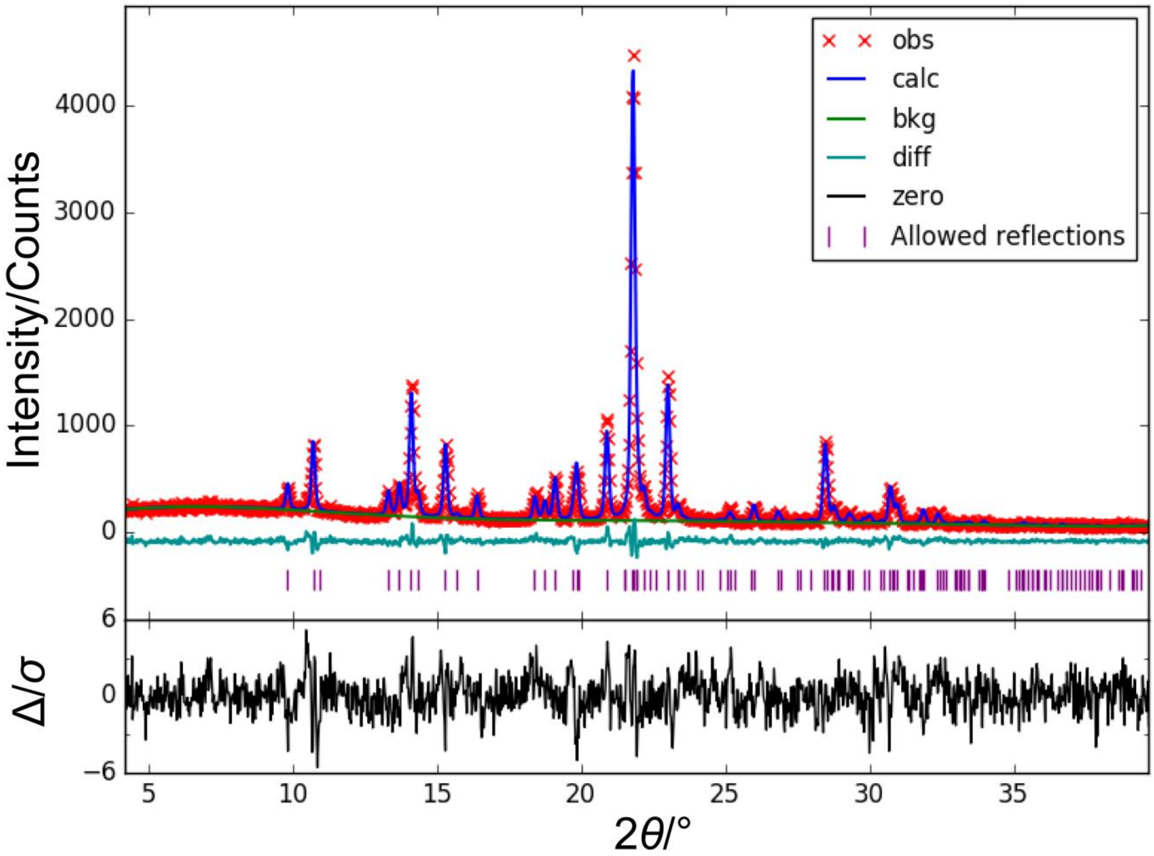

Fig S19. Final observed (red crosses) and calculated (blue line) profiles for the Rietveld refinement of monohydrate of **6**. The difference between both [(yobs − ycalc)] is marked as a dark cyan solid line, and weighted difference [(yobs − ycalc)/σ] as black solid line. Purple ticks mark position of allowed reflections. The PXRD data used for crystal-structure determination was collected for sample of **6** after slurry.

Table S2. The intermolecular interactions present in the crystal structures of compounds **1-11**.

Compound	Total number of intermolecular interactions per molecule	Type of intermolecular interaction	$d_{A–H\cdots B}$ (Å)	$D_{A\cdots B}$ (Å)	$\angle_{A–H\cdots B}$ (°)
**2** (PEXXEW)	10/6[b]	C–H···N	2.70<br>2.56<br>2.83<br>2.79	3.49<br>3.44<br>3.49<br>3.68	144.2<br>159.0<br>127.9<br>160.0
	4	C–H··· π_{Ar} (edge-to-face)	2.65<br>2.84	3.53<br>3.63	159.0<br>143.3
	2	π-π stacking (face-to-face)	-<br>-	3.65<br>3.73	-<br>-
**2**·H_2O (HIRLUQ)	2	O–H···N	2.20<br>2.16	3.01<br>2.97	158.4<br>158.3
	1	C–H···O	2.48	3.31	148.0
	4	C–H···N	2.70<br>2.75	3.56<br>3.57	153.5<br>147.6
	2	C–H··· π_{Ar} (edge-to-face)	2.97	3.63	128.9
**2**·4H_2O (OXUHIA02)	4/1[f]	O–H···N	2.08<br>2.24	2.87<br>2.98	163.9<br>168.1
	6/2/1[g]	C–H···O	2.72<br>2.69<br>2.78	3.58<br>3.58<br>3.50	155.1<br>159.8<br>134.9
	2	O–H···O	2.11<br>1.98	2.84<br>2.80	153.5<br>167.0
	8	C–H··· π_{Ar} (edge-to-face	3.10<br>3.31	3.70<br>3.78	122.9<br>113.9
**3**·3H_2O (OXUJAU02)	2/1/0[h]	O–H···N	2.07<br>1.90<br>-<br>-	2.91<br>2.85<br>2.84[c]<br>2.82[c]	166.5<br>167.4<br>-<br>-
	3/2/4[d]	O–H···O	1.95<br>1.90<br>1.93<br>1.80<br>-<br>-<br>-	2.86<br>2.77<br>2.84<br>2.78<br>2.96[c]<br>2.99[c]<br>3.00[c]	172.8<br>166.1<br>169.2<br>179.2<br>-<br>-<br>-
	4	C–H···N	2.45	3.38	166.9

			2.70	3.63	167.2
			2.53	3.46	165.6
			2.73	3.67	172.2
	2/1/0[i]	C–H···O	2.41	3.29	154.1
			2.54	3.45	159.6
			2.37	3.28	160.4
			2.36	3.28	164.1
	7	π_{Ar}-π_{Ar} stacking (face-to-face)	-	3.58	-
			-	3.48	-
			-	3.81	-
			-	3.64	-
			-	3.75	-
			-	3.70	-
			-	3.50	-
**4** (RUYKIF)	8	C–H···N	2.66	3.37	132.0
			2.84	3.59	136.5
	4	π-π stacking (face-to-face)	-	3.56	-
**5**·xH_2O[a] (MINXIT)	1	O–H···N	2.01/2.24[e]	2.86/2.88[e]	166.2/130.3[e]
			2.02/1.94[e]	2.89/2.77[e]	177.7/160.4[e]
	2	O–H···O	1.61	2.46	166.6
			1.91/1.63[e]	2.77/2.48[e]	172.1/166.5[e]
	2/0[b]	C–H···N	2.75	3.63	155.3
	1/0[b]	C–H···O	2.71	3.56	149.7
	6	π_{Ar}-π_{Ar} stacking (face-to-face)	-	3.80	-
**6** (MINWAK)	14	C–H···N	2.72	3.63	166.2
			2.65	3.56	163.7
			2.98	3.53	119.7
			2.81	3.54	136.1
			2.82	3.48	128.3
			2.96	3.87	168.7
			2.73	3.61	158.9
	2	C–H··· π	2.71	3.47	139.1
**6·$H_2$0**	2	O–H···N	1.98	2.74	144.9
			2.24	3.05	160.8
	2	C–H···O	2.64	3.47	149.9
			2.44	3.35	165.0
	1	π-π stacking (face-to-face)	-	3.60	-
	2	C–H··· π	2.990	3.83	151.2
**7**·4H_2O	2/1/0[k]	O–H···N	2.15	2.98	164.6
	2	O-H···O	1.83	2.66	163.3

			2.36	3.05	139.1
	4/2/0[j]	C–H···O	2.52	3.40	158.6
			2.69	3.56	156.2
	4	π-π stacking (face-to-face)	-	3.91	-
**8** (XAPTEO)	6	C–H···N	2.56	3.39	145.8
			2.78	3.52	136.0
			2.67	3.55	154.6
	4	C–H··· π_{Ar} (edge-to-face)	2.91	3.51	122.5
			2.95	3.73	140.7
**8** (MINVUD)	2	C–H··· π_{Ar} (edge-to-face)	2.91	3.36	111.6
	4	C–H··· N	2.82	3.61	143.4
	4	π-π stacking (face-to-face)	-	3.87	-
**9** (MINWEO)	18/10[b]	C–H···N	2.73	3.59	150.3
			2.85	3.44	121.3
			2.81	3.48	128.0
			2.89	3.73	148.8
			2.89	3.59	131.9
			2.86	3.74	154.2
			2.87	3.57	131.4
	28	C–H··· π_{Ar} (edge-to-face)	3.21	3.68	112.9
			2.85	3.65	142.4
			3.05	3.69	126.0
			3.04	3.56	116.1
			2.62	3.38	137.5
			2.69	3.49	143.1
			2.74	3.61	151.3
**10**	12	C–H···N	2.56	3.48	162.7
			2.81	3.55	134.8
			2.73	3.64	160.3
			2.67	3.58	160.5
			2.66	3.46	141.6
			2.65	3.58	167.5
	8	π-π stacking (face-to-face)	-	3.74	-
**11** (OLEPOK)	8	C–H···N	2.70	3.56	152.8
			2.84	3.71	156.6
	4	π-π stacking (face-to-face)	-	3.84	-
**11** (OLEPOK01)	8	C–H···N	2.58	3.50	160.5
			2.61	3.47	153.8
			2.67	3.52	144.7
			2.69	3.52	144.6

8	π-π stacking (face-to-face)	-	3.74	-

[a] The metrics for intermolecular interactions for disordered water molecules could not be measured.

[b] Each symmetrically independent molecule participates in different number of interactions.

[c] Position of hydrogen atoms of water molecules not determined.

[d] Different symmetrically independent water molecules participate in different number of interactions; one water molecule is involved in four contacts, two are involved in three contacts, and three are involved in two contacts.

[e] Due to the disorder of water molecules two sets of values for each O-H···N contact were given.

[f] Due to the 4:1 water:compound **2** ratio, number of O-H···N contact per molecule of each component differ; each molecule of **2** is involved in 4 such contacts, while each molecule of water is involved in one such contact.

[g] Due to the 4:1 water:compound **2** ratio, and 2 water molecules in asymmetric part of the unit cell number of C-H···O contact per molecule of each component, and each asymmetric water molecule, differ: each molecule of **2** is involved in 6 such contacts, two molecule of water are involved in two such contact each, and two others in one each.

[h] Due to the 11:4 water: compound **3** ratio, as well as presence of 2 molecules of **3** and 5.5 molecules of water in the asymmetric part of the unit-cell, molecules are involved in different number of O-H···N contacts; each molecule of **3**, as well as one water molecule, are involved in 2 such bonds, 2 water molecules are involved in 1 such bond each, and 3 water molecules are not involved at all.

[i] Each molecule of **3** is involved in two C-H···O contacts, four water molecules are involved in one such contact each, and two water molecules do not form C-H···O contacts at all.

[j] Each molecule of **7** is involved in four C-H···O contacts, two water molecules are involved in two such contact each, and two water molecules do not form C-H···O contacts at all.

[k] Each molecule of **7** is involved in two O-H···N contacts, two water molecules are involved in one such contact each, and two water molecules do not form O-H···N contacts at all.

Note: The centroids of the π-systems were created in order to measure the bond distances and angles of the corresponding interactions.

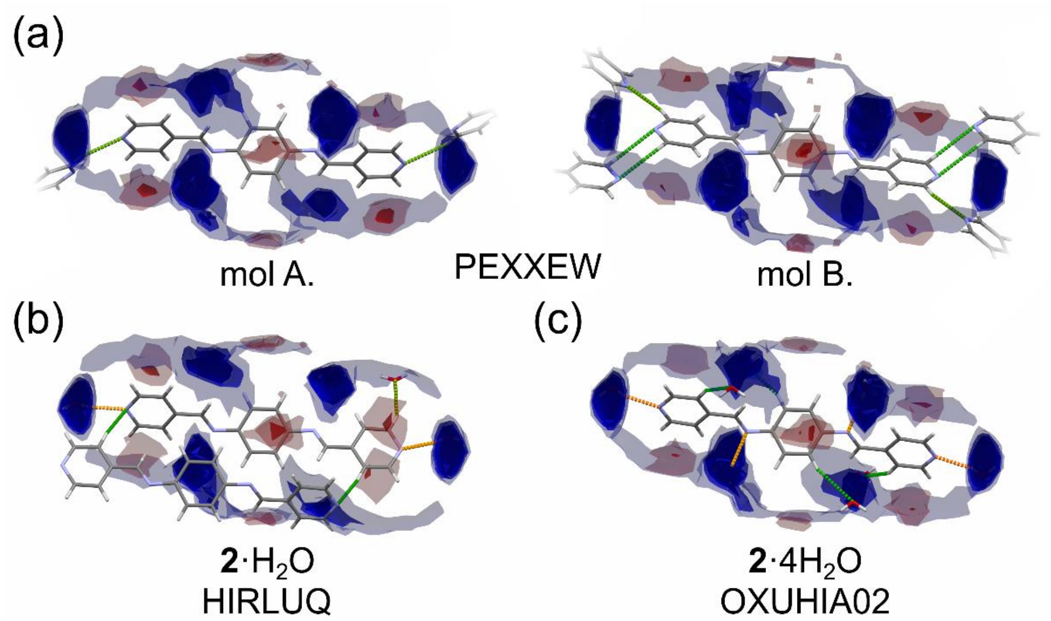

Fig S20. Full Interaction Maps (FIMs) for the molecular conformers present in crystal structures of 2: (a) anhydrate (CSD refcode PEXXEW), (b) monohydrate (HIRLUQ) and (c) tetahydrate (OXUHIA02). The blue and red contours indicate regions most commonly taken by hydrogen-bond-involved water molecule and aromatic C-H moieties, respectively. The opacity of the region is positively corelated to the probability of the interaction existence. A color scale (from green, through orange, to red) was applied for hydrogen bonds to mark their relative length (green being the longest and red being the shortest).

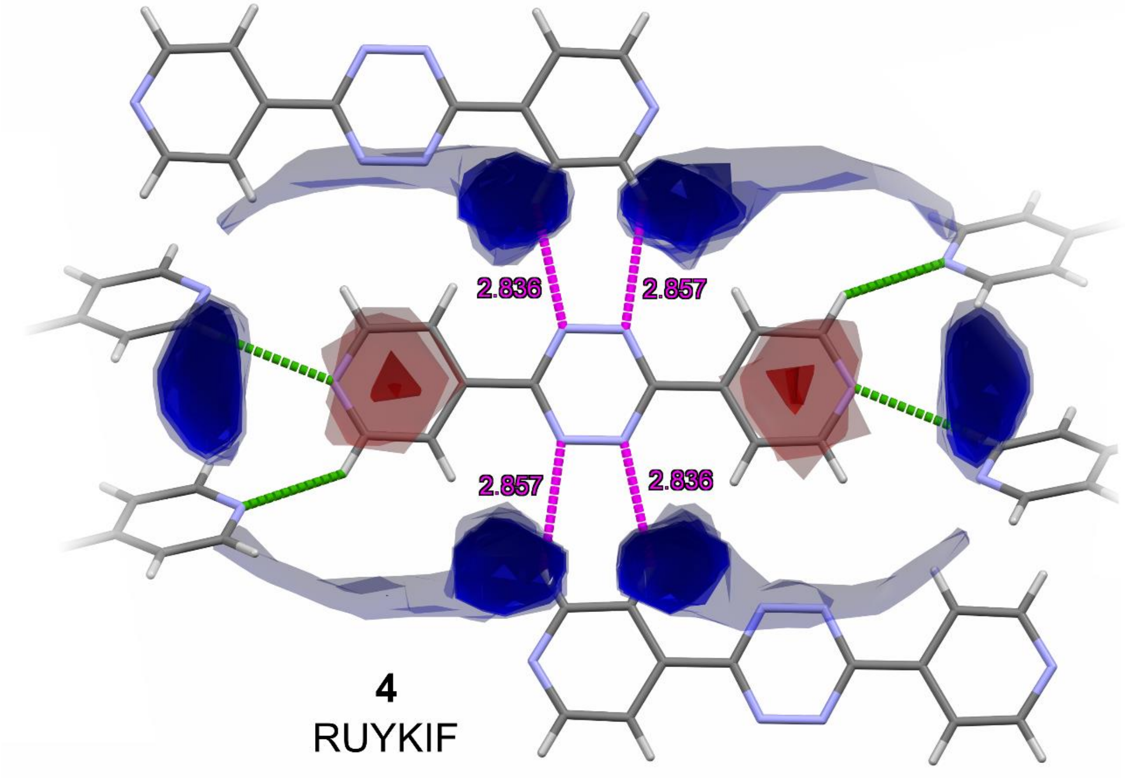

Fig S21. FIMs for anhydrate molecule 4, RUYKIF. For the color coding see Figure S20 caption. Possible very long contacts are marked in magenta.

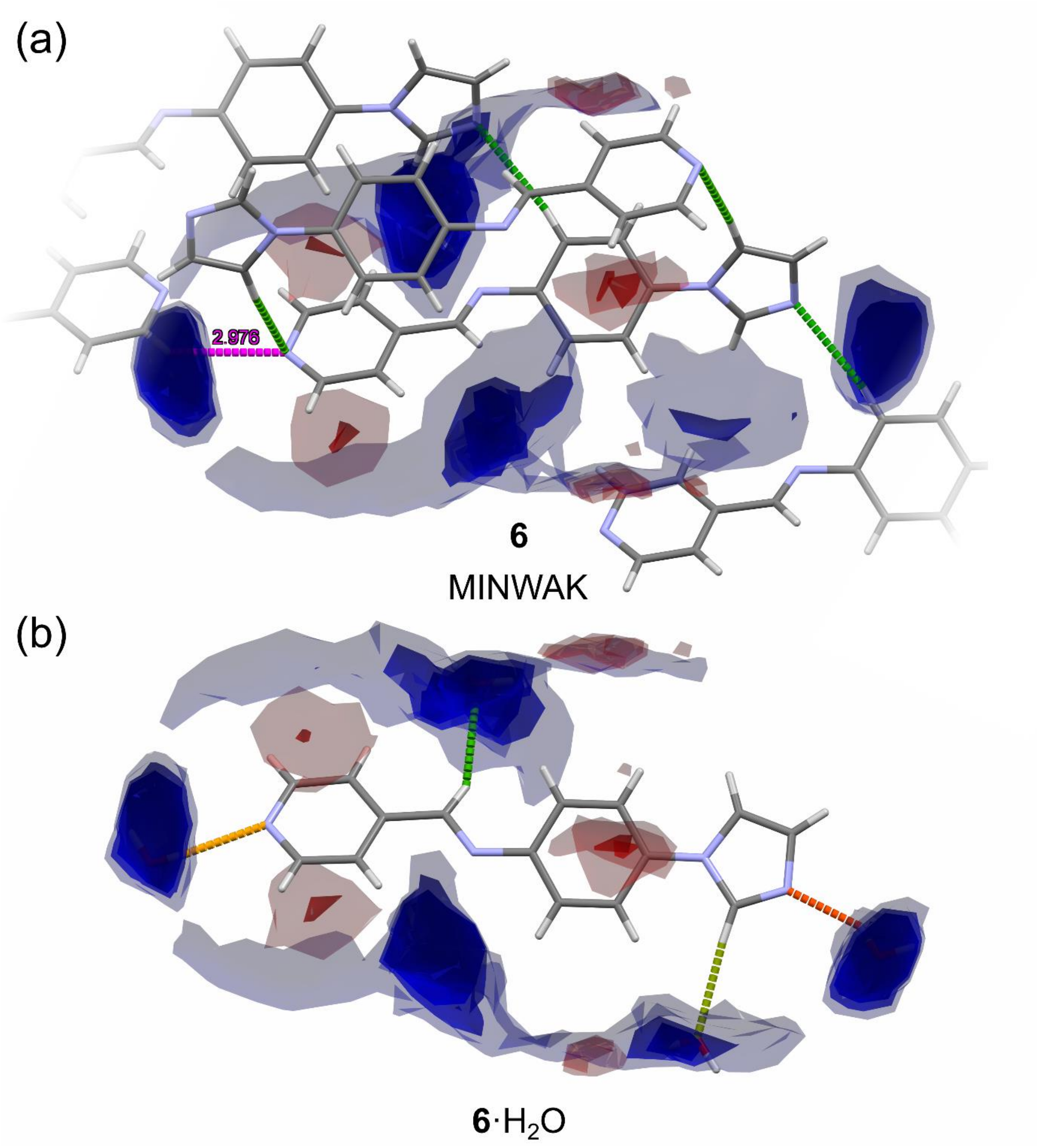

Fig S22. Interaction preferences for the molecular conformation of (a) anhydrate (MINWAK) and (b) hydrate forms of 6. For a detailed description of the color coding see Figure S20 caption. Possible very long contacts are marked in magenta.

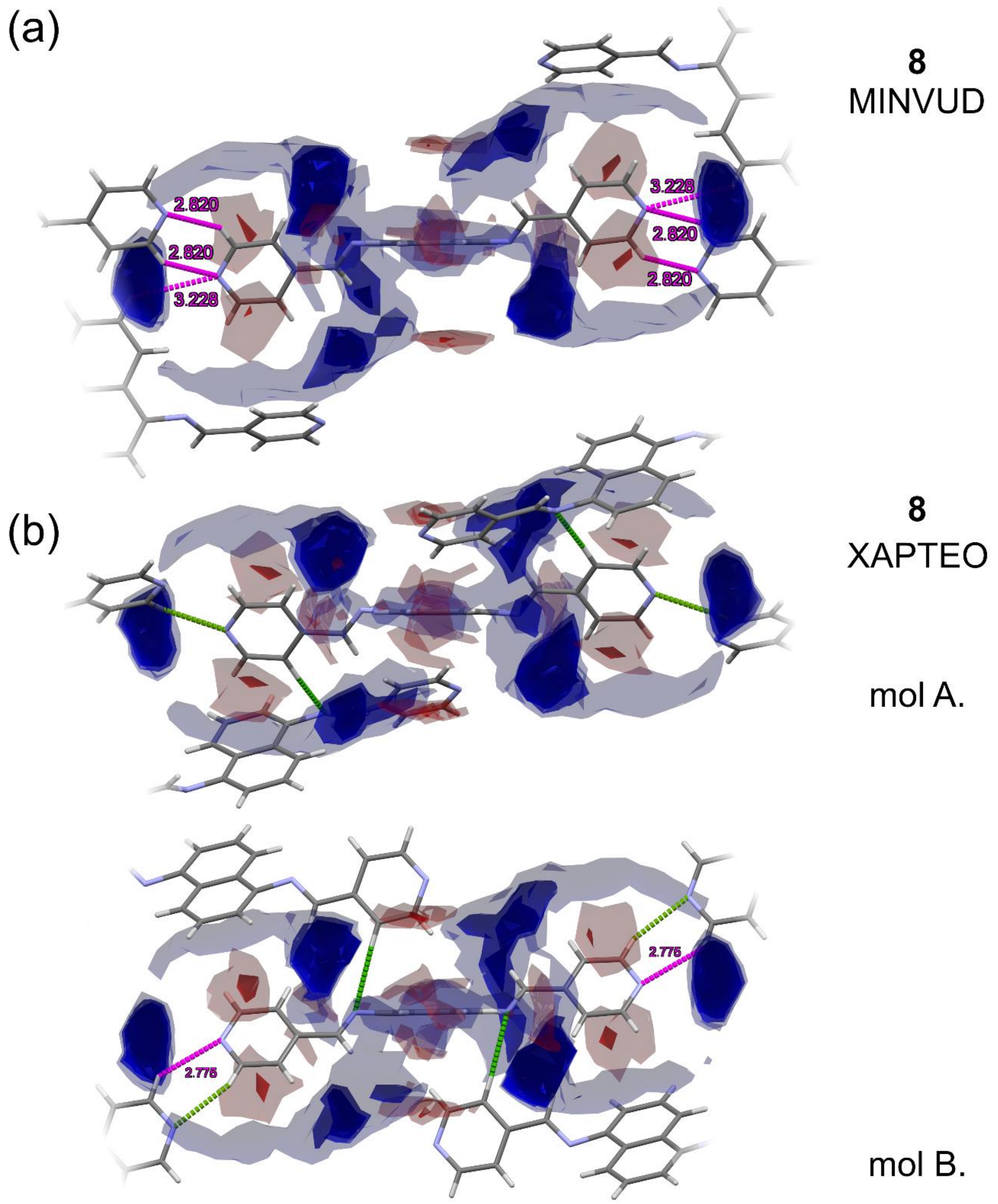

Fig S23. FIMs for anhydrate molecules 8, MINVUD (a) and XAPTEO (b). For the color coding see Figure S20 caption. Possible very long contacts are marked in magenta.

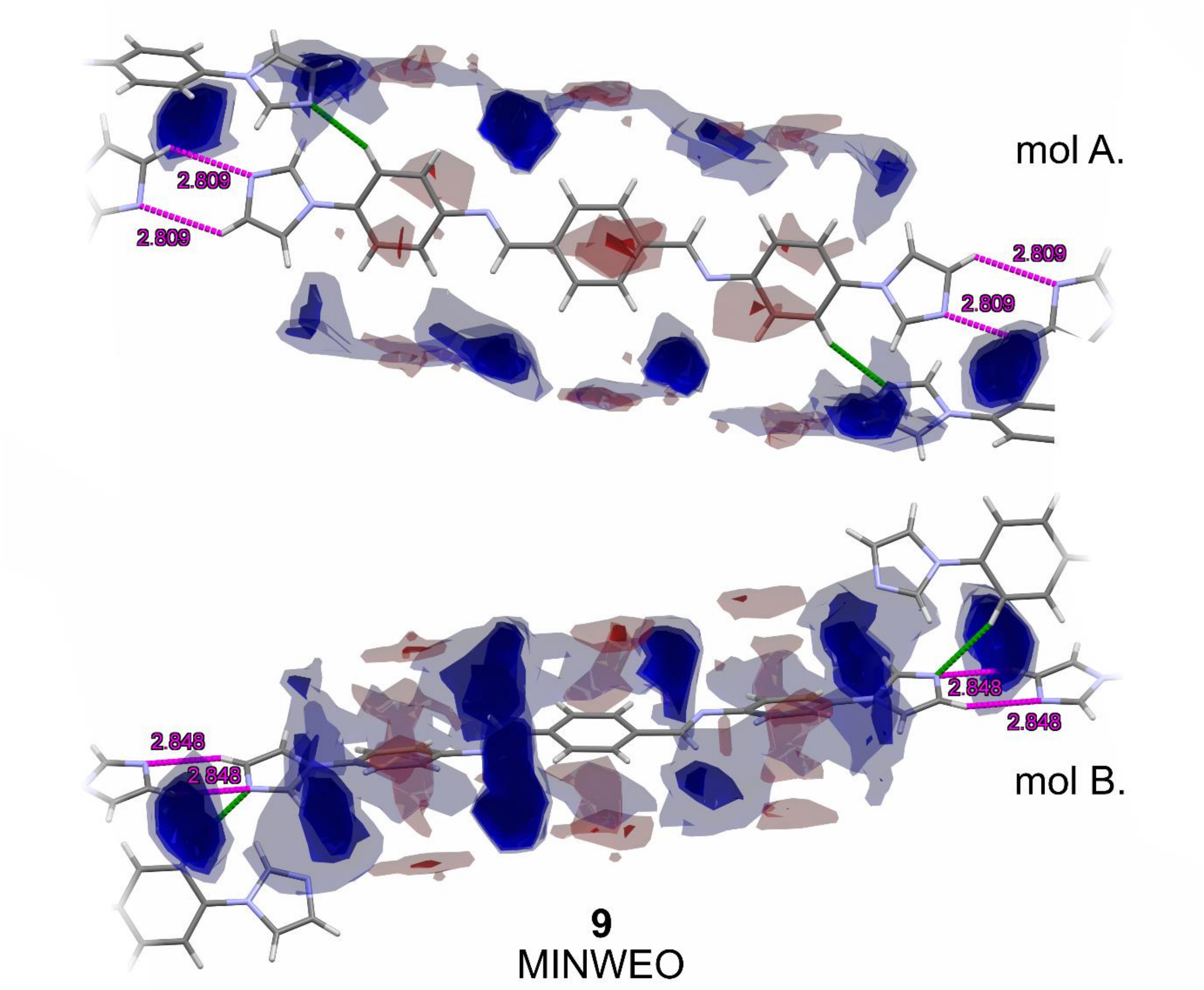

Fig S24. FIMs for anhydrate molecule 9 (MINWEO). For the color coding see Figure S20 caption. Possible very long contacts are marked in magenta.

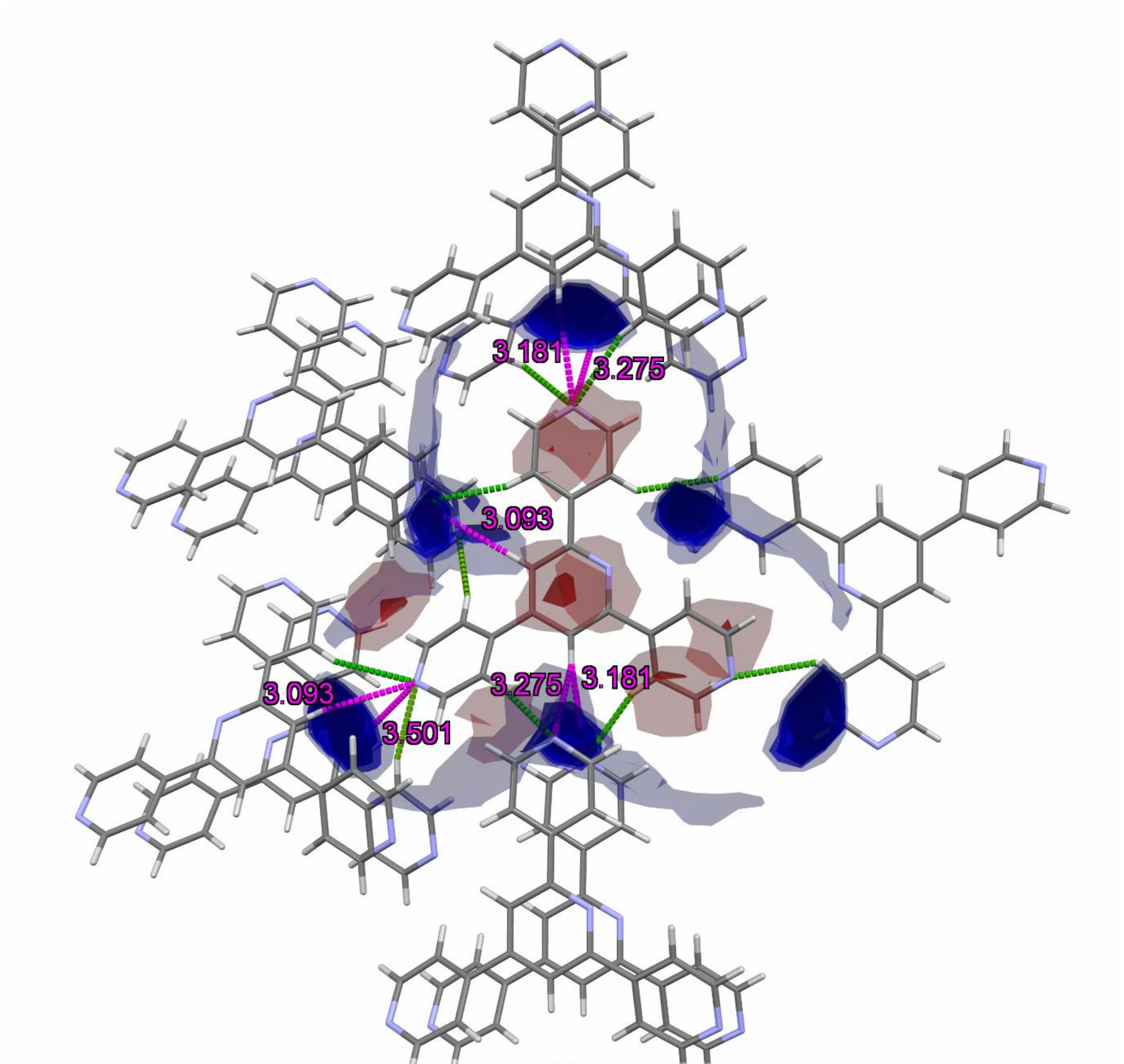

10

Fig S25. FIMs for anhydrate molecule 10. For the color coding see Figure S20 caption. Possible very long contacts are marked in magenta.

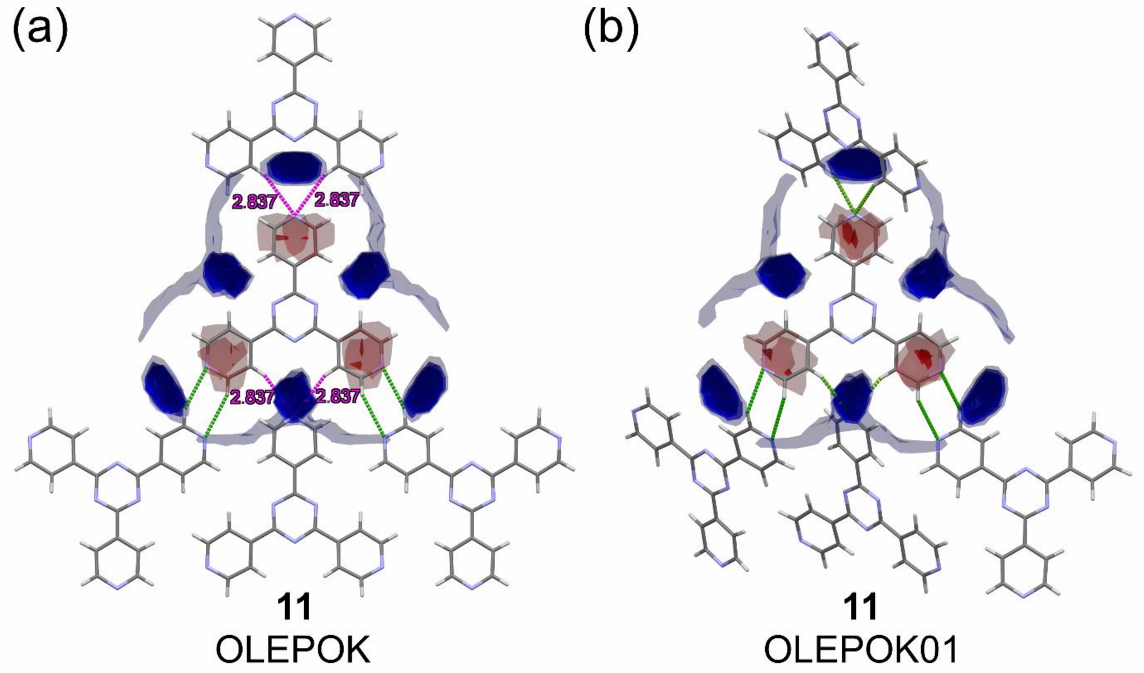

Fig S26. FIMs for anhydrate molecules 11, OLEPOK (a) and OLEPOK01 (b). For the color coding see Figure S20 caption. Possible very long contacts are marked in magenta.

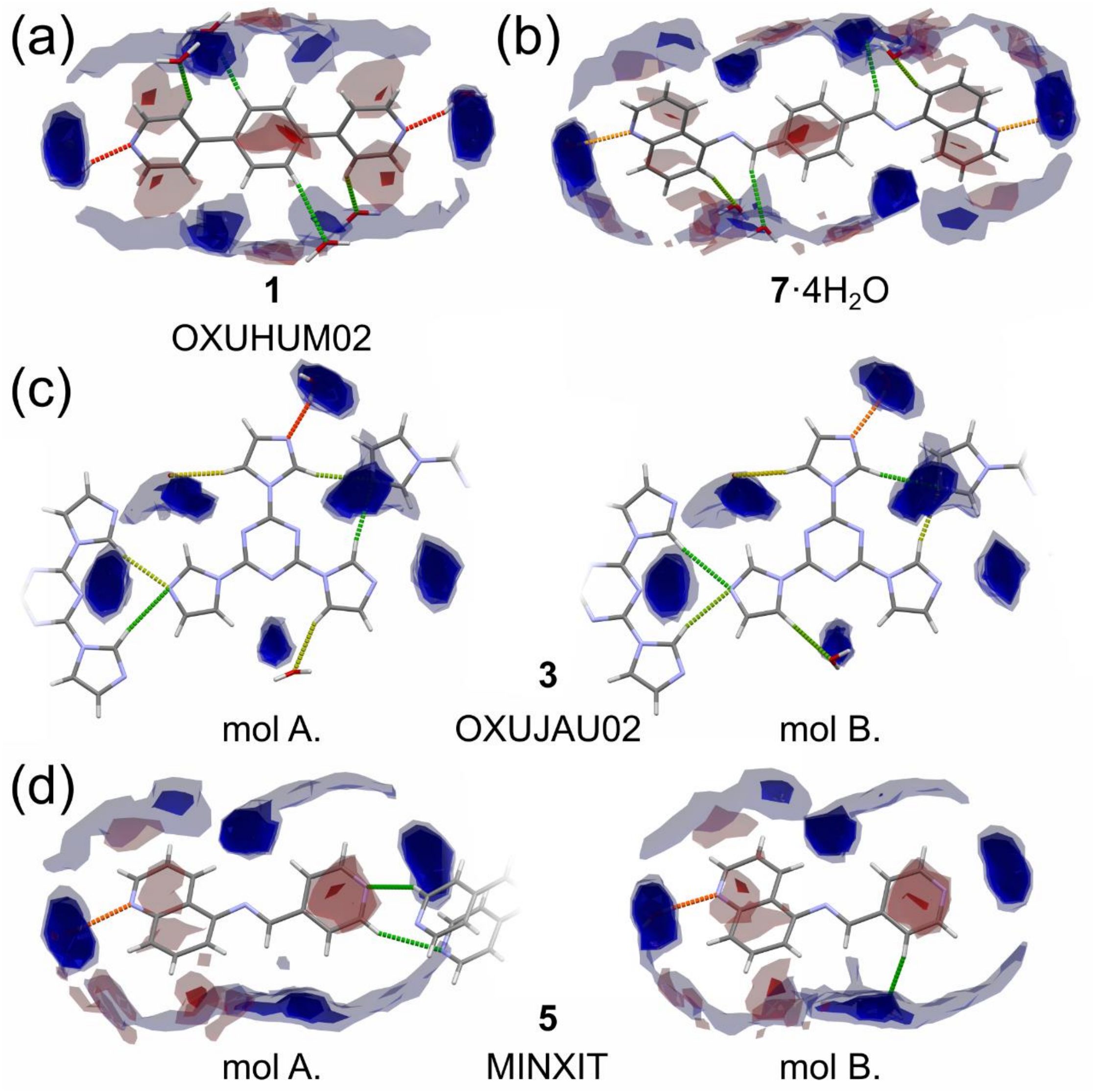

Fig S27. FIMs for anhydrate molecules 1 (OXUHUM02) (a), 7 (b), 3 (OXUJAU02) (c) and 5 (d). For the color coding see Figure S20 caption.

Table S3. List of dihedral and torsion angles observed in the crystal structures of compounds **1-11**.

Compound	Structure code	Torsion angles between aryl rings (°)						Torsion angles about imine fragment (°)	
		∠AB	∠BC	∠AC	∠AD	∠BD	∠DC	C^1-C^2-C^3-N^4	C^3-N^4-C^5-C^6
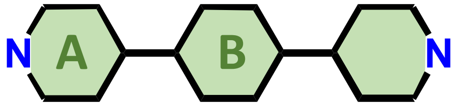 **1**	**OXUHUM02**	31.65	-	-	-	-	-	-	-
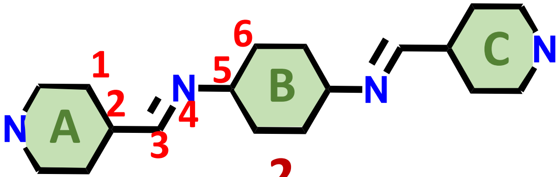 **2**	**PEXXEW**	53.29, 58.10[a]	-	-	-	-	-	9.98(6), 6.10(6)[a]	47.61(5), 47.87(5)[a]
	**OXUHIA02**	52.49	-	-	-	-	-	8.74(1)	42.87(1)
	**HIRLUQ**	24.96	43.79	68.60	-	-	-	4.64(2), 4.54(2)[b]	28.72(2), 40.58(2)[b]
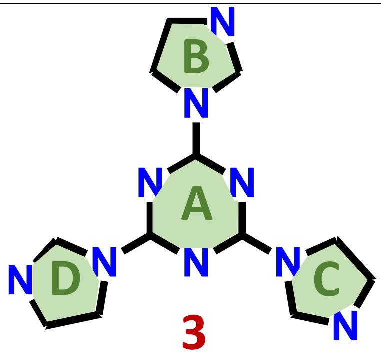 **3**	**OXUJAU02**	4.03, 1.93[b]	6.72, 3.82[b]	3.10, 2.62[b]	11.31, 4.46[b]	8.31 5.11[b]	14.14, 6.84	-	-

Compound	Structure code	Torsion angles between aryl rings (°)						Torsion angles about imine fragment (°)	
		∠AB	∠BC	∠AC	∠AD	∠BD	∠DC	C^1-C^2-C^3-N^4	C^3-N^4-C^5-C^6
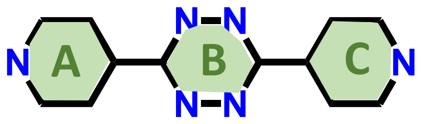 4	**RUYKIF**	2.56	2.56	-	-	-	-	-	-
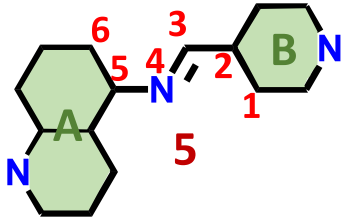 5	**MINXIT**	37.99, 41.55[a]	-	-	-	-	-	0.40(1), 0.78(1)[a]	142.06(1), 144.19(1)[a]
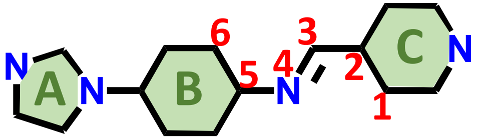 6	**MINWAK**	40.61	39.15	79.75	-	-	-	4.86(1)	45.39(1)
	**H_2O : New**	6.45	35.43	29.03	-	-	-	3.64(1)	31.85(1)

Compound	Structure code	Torsion angles between aryl rings (°)						Torsion angles about imine fragment (°)	
		∠AB	∠BC	∠AC	∠ AD	∠ BD	∠ DC	C^1-C^2-C^3-N^4	C^3-N^4-C^5-C^6
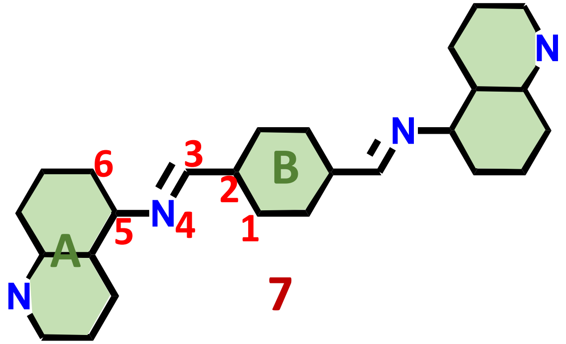 7	MINMOO	42.51	-	-	-	-	-	1.65(1)	142.55(1)
	4H₂O : New	42.09	-	-	-	-	-	2.70(1)	39.90(1)
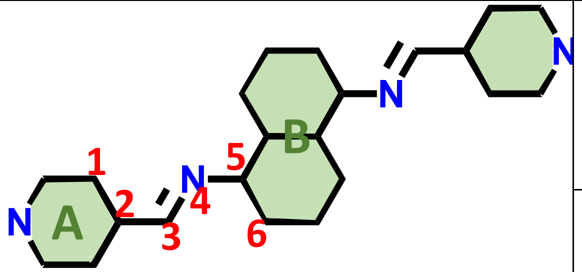 8	MINVUD	62.52	-	-	-	-	-	13.40(1)	132.18(1)
	XAPTEO01	57.52, 53.85[a]	-	-	-	-	-	1.14(1), 6.78(1)[a]	129.05(1), 133.32(1)[a]
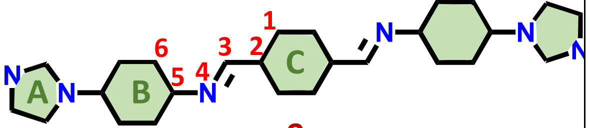 9	MINWEO	25.80 24.62[a]	18.32 16.00[a]	15.35 10.86[a]	-	-	-	167.70(4), 170.88(5)[a]	28.21(8), 24.41(8)[a]

Compound	Structure code	Torsion angles between aryl rings (°)						Torsion angles about imine fragment (°)	
		∠AB	∠BC	∠AC	∠ AD	∠ BD	∠ DC	C^{1}-C^{2}-C^{3}-N^{4}	C^{3}-N^{4}-C^{5}-C^{6}
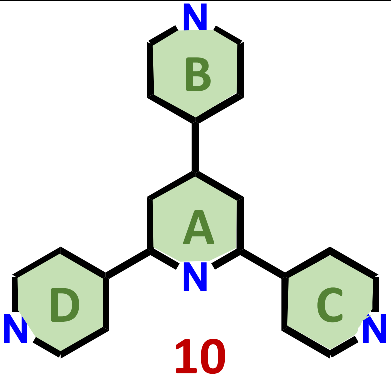 10	**Anh : New**	38.98	-	14.23	18.08	-	-	-	-
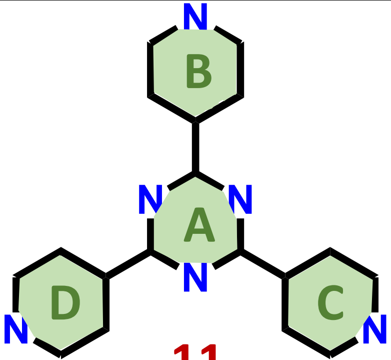 11	**OLEPOK**	6.85	-	6.85	8.08	-	-	-	-
	**OLEPOK01**	4.31	-	1.46	1.46	-	-	-	-

[a]For these crystal structures, molecules of respective compounds lie on a centre of symmetry. However, there are two halves of crystallographically independent molecules in the asymmetric unit, therefore two sets of values are listed in the table.

[b]In this crystal structure, the molecule lies on a general position; therefore, it is non-symmetrical and therefore two sets of values are included in the table.

References:

(1) Duggirala, N. K.; Perry, M. L.; Almarsson, Ö.; Zaworotko, M. J. Pharmaceutical Cocrystals: Along the Path to Improved Medicines. *Chem. Commun.* **2016,** *52* (4), 640.

(2) Healy, A. M.; Worku, Z. A.; Kumar, D.; Madi, A. M. Pharmaceutical Solvates, Hydrates and Amorphous Forms: A Special Emphasis on Cocrystals. *Adv. Drug Delivery Rev.* **2017,** *117*, 25.

(3) Aitipamula, S.; Banerjee, R.; Bansal, A. K.; Biradha, K.; Cheney, M. L.; Choudhury, A. R.; Desiraju, G. R.; Dikundwar, A. G.; Dubey, R.; Duggirala, N.et al. Polymorphs, Salts, and Cocrystals: What's in a Name? *Cryst. Growth Des.* **2012,** *12* (5), 2147.

(4) Giron, D.; Goldbronn, C.; Mutz, M.; Pfeffer, S.; Piechon, P.; Schwab, P. Solid State Characterizations of Pharmaceutical Hydrates. *J. Therm. Anal. Calorim.* **2002,** *68* (2), 453.

(5) Gardner, C. R.; Walsh, C. T.; Almarsson, Ö. Drugs as Materials: Valuing Physical Form in Drug Discovery. *Nat. Rev. Drug Discov.* **2004,** *3* (11), 926.

(6) Variankaval, N.; Cote, A. S.; Doherty, M. F. From Form to Function: Crystallization of Active Pharmaceutical Ingredients. *AIChE J.* **2008,** *54* (7), 1682.

(7) Gorbitz, C. H.; Hersleth, H.-P. On the Inclusion of Solvent Molecules in the Crystal Structures of Organic Compounds. *Acta Crystallogr. Sect. B: Struct. Sci.* **2000,** *B56* (3), 526.

(8) Morris, K. R.; Rodriguez-Hornedo, N. *Hydrates, Encyclopedia of Pharmaceutical Technology*; J. Swarbrick and J. C. Boylan ed.: New York: Marcel Dekker, Inc., 1993.

(9) Gillon, A. L.; Feeder, N.; Davey, R. J.; Storey, R. Hydration in Molecular Crystals A Cambridge Structural Database Analysis. *Cryst. Growth Des.* **2003,** *3* (5), 663.

(10) Desiraju, G. R. Hydration in Organic Crystals: Prediction from Molecular Structure. *J. Chem. Soc., Chem. Commun.* **1991,** (6), 426.

(11) Morris, K. R. *Structural Aspects of Hydrates and Solvates*; Brittain, H. G. ed.; New York: Marcel Dekker, Inc., : In Polymorphism in Pharmaceutical Solids, 1999.

(12) Pudipeddi, M.; Serajuddin, A. T. M. Trends in Solubility of Polymorphs. *J. Pharm. Sci.* **2005,** *94* (5), 929.

(13) Newman, A. W.; Reutzel-Edens, S. M.; Zografi, G. Characterization of the "Hygroscopic" Properties of Active Pharmaceutical Ingredients. *J. Pharm. Sci.* **2008,** *97* (3), 1047.

(14) Ahlneck, C.; Zografi, G. The Molecular Basis of Moisture Effects on the Physical and Chemical Stability of Drugs in the Solid State. *Int. J. Pharm.* **1990,** *62* (2), 87.

(15) Khankari, R. K.; Grant, D. J. W. Pharmaceutical Hydrates. *Thermochim. Acta,* **1995,** *248*, 61.

(16) Byrn, S. R.; Zografi, G.; Chen, X. S. *Solvates and Hydrates*; 1st ed.; John Wiley & Sons, Inc.: Hoboken, NJ, USA,: in Solid State Properties of Pharmaceutical Materials, 2017.

(17) Hickey, M. B.; Peterson, M. L.; Manas, E. S.; Alvarez, J.; Haeffner, F.; Almarsson, Ö. Hydrates and Solid-State Reactivity: A Survey of β-Lactam Antibiotics. *J. Pharm. Sci.* **2007,** *96* (5), 1090.

(18) Vippagunta, S. R.; Brittain, H. G.; Grant, D. J. Crystalline Solids. *Adv. Drug. Delivery Rev.* **2001,** *48* (1), 3.

(19) Carstensen, J. T. Effect of Moisture on the Stability of Solid Dosage Forms. *Drug Dev. Ind. Pharm.* **1988,** *14* (14), 1927.

(20) Giron, D. Thermal Analysis and Calorimetric Methods in the Characterisation of Polymorphs and Solvates. *Thermochim. acta* **1995,** *248*, 1.

(21) Xu, Y.; Jiang, L.; Huang, Y.; Wang, J. R.; Mei, X. Solid-State Characterization and Transformation of Various Creatine Phosphate Sodium Hydrates. *J. Pharm. Sci.* **2014,** *103* (11), 3688.

(22) Sear, J. W.; Hand, C. W.; Moore, R. A.; McQuay, H. J. Studies on Morphine Disposition: Influence of General Anaesthesia on Plasma Concentrations of Morphine and its Metabolites. . *Br. J. Anaesth.* **1989,** *62* (1), 22.

(23) Gandhi, R.; Pillai, O.; Thilagavathi, R.; Gopalakrishnan, B.; Kaul, C. L.; Panchagnula, R. Characterization of Azithromycin hydrates. *Eur. J. Pharm. Sci.* **2002,** *16* (3), 175.

(24) Rose, H. A. Erythromycin and Some of Its Derivatives. *Anal. Chem.* **1954,** *26* (5), 938.

(25) Lee, A. Y.; Erdemir, D.; Myerson, A. S. Crystal Polymorphism in Chemical Process Development. *Annu. Rev. Chem. and Biomol. Eng.* **2011,** *2* (1), 259.
(26) Gyseghem, E. V.; Stokbroekx, S.; de Armas, H. N.; Dickens, J.; Vanstockem, M.; Baert, L.; Rosier, J.; Schueller, L.; Van den Mooter, G. Solid State Characterization of the anti-HIV Drug TMC114: Interconversion of Amorphous TMC114, TMC114 Ethanolate and Hydrate. *Eur. J. Pharm. Sci.* **2009,** *38* (5), 489.
(27) Fujii, K.; Uekusa, H.; Itoda, N.; Yonemochi, E.; Terada, K. Mechanism of Dehydration–Hydration Processes of Lisinopril Dihydrate Investigated by ab Initio Powder X-ray Diffraction Analysis. *Cryst. Growth Des.* **2012,** *12* (12), 6165.
(28) Martinez, H.; Byrn, S. R.; Pfeiffer, R. R. Solid-State Chemistry and Crystal Structure of Cefaclor Dihydrate. *Pharm. Res.* **1990,** *7* (2), 147.
(29) Bahal, S. M., 1975; Vol. US Patent 3867523 A.
(30) Stephenson, G. A.; Groleau, E. G.; Kleemann, R. L.; Xu, W.; Rigsbee, D. R. Formation of Isomorphic Desolvates: Creating a Molecular Vacuum. *J. Pharm. Sci.* **1998,** *87* (5), 536.
(31) Bouzard, D.; Weber, A.; Stemer, J., 1985; Vol. US4504657.
(32) Agbada, C. O.; York, P. Dehydration of Theophylline Monohydrate Powder: Effects of Particle Size and Sample Weight. *Int. J. Pharm.* **1994,** *106* (1), 33.
(33) Sun, C.; Zhou, D.; Grant, D. J. W.; Young, V. G., Jr. Theophylline Monohydrate. *Acta Crystallogr. Sect. E: Struct. Rep. Online* **2002,** *58* (4), o368.
(34) Cazer, F. D.; Kane, M. J.; Scott, B. L.; Shahi, V., 1994; Vol. US5332832 A.
(35) Barnes, R. D.; Wood-Kaczmar, M. W.; Curzons, A. D.; Lynch, I. R.; Richardson, J. E.; Buxton, P. C., 1988; Vol. US4721723.
(36) Brittain, H. G.; Morris, K. R.; Boerrigter, S. X. M. *Structural Aspects of Solvatomorphic Systems in Polymorphism in Pharmaceutical Solids*; 2nd ed.; Informa Healthcare: New York, 2009.
(37) Henck, J. O.; Griesser, U. J.; Burger, A. Polymorphism of Drug Substances : An Economic Challenge. *Pharm. Ind.* **1997,** *59*, 165.
(38) Griesser, U. J. *The Importance of Solvates.*; Hilfiker, R. ed.; Weinheim, Germany: Wiley-VCH,Verlag GmbH and Co.: In Polymorphism in the Pharmaceutical Industry, 2006.
(39) Rodríguez-Spong, B.; Price, C. P.; Jayasankar, A.; Matzger, A. J.; Rodríguez-Hornedo, N. r. General Principles of Pharmaceutical Solid Polymorphism: A Supramolecular Perspective. *Adv. Drug Deliv. Rev.* **2004,** *56* (3), 241.
(40) Bajpai, A.; Scott, H. S.; Pham, T.; Chen, K.-J.; Space, B.; Lusi, M.; Perry, M. L.; Zaworotko, M. J. Towards an Understanding of the Propensity for Crystalline Hydrate Formation by Molecular Compounds. *IUCrJ* **2016,** *3* (6), 430.
(41) Sanii, R.; Bajpai, A.; Patyk-Kaźmierczak, E.; Zaworotko, M. J. High Yield, Low-Waste Synthesis of a Family of Pyridyl and Imidazolyl-Substituted Schiff Base Linker Ligands. *ACS Sustain. Chem. Eng.* **2018,** *6* (11), 14589.
(42) Azarifar, D.; Zolfigol, M. A.; Forghaniha, A. A Convenient Method for the Preparation of Some New Derivatives of 1,3,5-s-Triazine under Solvent Free Condition. *Heterocycles* **2004,** *63* (8), 1897.
(43) Li, C.; Ge, H.; Yin, B.; She, M.; Liu, P.; Li, X.; Li, J. Novel 3,6-Unsymmetrically Disubstituted-1,2,4,5-Tetrazines: S-Induced one-Pot Synthesis, Properties and Theoretical Study. *RSC Adv.* **2015,** *5* (16), 12277.
(44) Chen, K. J.; Perry Iv, J. J.; Scott, H. S.; Yang, Q.-Y.; Zaworotko, M. J. Double-Walled pyr Topology Networks from a Novel Fluoride-Bridged Heptanuclear Metal Cluster. *Chem. Sci.* **2015,** *6* (8), 4784.
(45) Janczak, J.; Śledź, M.; Kubiak, R. Catalytic Trimerization of 2- and 4-Cyanopyridine Isomers to the Triazine Derivatives in Presence of Magnesium Phthalocyanine. *J. Mol. Struct.* **2003,** *659* (1), 71.
